# The Importance of Conserving the Stoichiometry of Wide-Bandgap Perovskites in Additive Engineering

**DOI:** 10.1021/acsaem.5c02216

**Published:** 2025-09-17

**Authors:** Nick R. M. Schipper, Guus J. W. Aalbers, Laura Bellini, Simon V. Quiroz Monnens, Lana M. Kessels, Junke Wang, Martijn M. Wienk, René A. J. Janssen

**Affiliations:** † Molecular Materials and Nanosystems & Institute for Complex Molecular Systems, 3169 Eindhoven University of Technology, P.O. Box 513, Eindhoven 5600 MB, The Netherlands; ‡ Clarendon Laboratory, Department of Physics, 6396 University of Oxford, Oxford OX1 3PU, U.K.; § Dutch Institute for Fundamental Energy Research, De Zaale 20, 5612 AJ, Eindhoven 5612, The Netherlands

**Keywords:** perovskite, stoichiometry, additive engineering, solar cells, device performance

## Abstract

Additive engineering is among the most commonly used strategies to enhance the performance and stability of perovskite solar cells. Prior research often focused on optimizing device performance by using additives in the perovskite precursor solution to influence the rate of crystallization and film formation, but a fundamental understanding of the effect of additives on the stoichiometry of the absorber remains elusive. In this study, we reveal how additives affect the ABX_3_ stoichiometry of the perovskite absorber and its photovoltaic properties. We find that the solar cell performance of a wide-bandgap (1.77 eV) Cs_0.2_FA_0.8_Pb­(I_0.6_Br_0.4_)_3_ perovskite decreases when processed with either of two common additives, lead thiocyanate and lead chloride, because the additive disturbs the stoichiometry. Interestingly, the addition of excess formamidinium iodide (FAI) to the precursor solution can restore the initial ABX_3_ stoichiometry and fully recover the device performance. The excess of FAI that is required depends on whether the halide or pseudohalide additive is incorporated into the crystal lattice. Finally, we alter the stoichiometry of an additive-free perovskite absorber by inducing either an excess or a deficiency of FAI or lead iodide in the precursor and show that slight deviations from the ideal stoichiometry rapidly degrade the device performance. This work provides fundamental insights into the importance of bulk stoichiometry in perovskite absorbers and can serve as a basis for future rational additive engineering.

## Graphical abstract

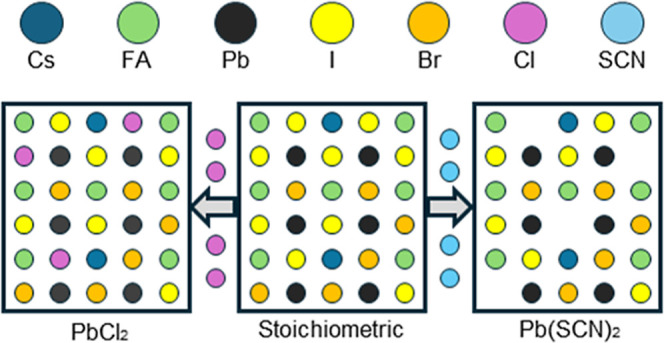

## 1 Introduction

Metal-halide perovskite solar cells have gained significant interest over the past decade, leading to a rapid increase of power conversion efficiency (PCE) from 3.8% for single-junction solar cells in 2009 to 34.85% for a perovskite-on-silicon tandem solar cell in 2025.^1,2^ Metal-halide perovskites are semiconductors with an ABX_3_ stoichiometry, in which A is a monovalent cation, such as formamidinium (FA^+^), cesium (Cs^+^), or methylammonium (MA^+^), B is a bivalent metal cation such as lead (Pb^2+^) or tin (Sn^2+^), and X is a monovalent halide anion, i.e., iodide (I^–^), bromide (Br^–^), or chloride (Cl^–^). By changing the chemical composition, the bandgap of metal-halide perovskites can be varied over a wide range, making them of special interest for multijunction solar cells. In these architectures, multiple absorbers with cascaded bandgaps are stacked and tuned to specific regions of the solar spectrum to reduce thermalization and absorption losses.^3^

Despite their potential and efforts aimed at improving the performance of single-junction perovskite solar cells to approach the Shockley–Queisser limit,^4^ metal-halide perovskites remain prone to conversion losses due to defects. Shallow defects located close to either the valence or conduction band act as temporary charge carrier trapping centers,^5^ while deep defects located in the middle of the bandgap are effective centers for charge carrier recombination.^6^ Defect states in the bulk or at interfaces contribute significantly to nonradiative charge recombination and are thus directly related to losses in open-circuit voltage (*V*_oc_). Examples of these defects include halide vacancies,^7^ interstitials,^8^ and antisites,^9^ but also grain boundaries can have a significant effect on the losses in a solar cell.^10^ These effects become much more significant when increasing the bandgap of the solar cell by replacing I^–^ with Br^–^ because the crystallization dynamics and formation energy of iodide- and bromide-rich perovskites differ significantly, for example leading to wrinkled films and halide segregation.^11^

In an effort to eliminate these defects, optimization strategies are employed that target either the perovskite bulk or adjacent interfaces. Examples include strain and lattice engineering,^12^ interface engineering,^13^ compositional engineering,^14^ and additive engineering,^15–18^ of which especially the latter has gained significant interest in recent years, due to a wide variety of additives that can enhance device performance.

Previously, Nguyen et al. showed that adding 2 mol % of lead thiocyanate (Pb­(SCN)_2_) into a 1.87 eV Cs_0.1_FA_0.9_PbI_1.4_Br_1.6_ perovskite precursor solution results in a 16-fold increase in grain size, accompanied by higher photoluminescence quantum yield due to a decrease in nonradiative recombination.^19^ The authors provided two parts of additional formamidinium iodide (FAI) per part of Pb­(SCN)_2_ to compensate for the FAI that is consumed while forming volatile formamidine and thiocyanic acid. Similar effects on the grain size were observed by Ke et al. in a 1.57 eV MAPbI_3_ composition, where 5 mol % of Pb­(SCN)_2_ resulted in a significant increase of the average grain size of up to 20 times, but without compensating with additional FAI.^20^ Likewise, lead thiocyanate has been found to increase surface uniformity and grain size in a tin-based FASnI_3_ perovskite.^21^

Lead chloride (PbCl_2_) is another commonly used additive, which is reported to retard perovskite crystallization and improve the crystallinity by forming an intermediate MAPbCl_3_ phase in MAPbI_3_ perovskites, and therewith suppresses charge recombination and enhances charge extraction.^22,23^ Furthermore, Zhang et al. showed that adding 10 mol % of PbCl_2_ into a MAPbI_3_ perovskite improves the perovskite film quality and significantly increases the grain size.^24^

While these prior studies report the beneficial effect on crystallinity and grain size by incorporating Pb­(SCN)_2_ or PbCl_2_ in the perovskite precursor solution, the role of these additives in the final film is less clear. In the present study, we propose that a universal mechanism governs lead-based additive engineering, which we ascribe to their capability to disturb the ABX_3_ stoichiometry of the perovskite absorber, depending on whether the halide or pseudohalide remains in the film after thermal annealing. We employ two commonly used additives, Pb­(SCN)_2_ and PbCl_2_, when forming a wide-bandgap (1.77 eV) Cs_0.2_FA_0.8_Pb­(I_0.6_Br_0.4_)_3_ perovskite. This bandgap is ideal for high-performing all-perovskite tandem solar cells, and serves as a model for a range of modern mixed-cation mixed-halide perovskites.^25^ Furthermore, this perovskite composition consists of most of the commonly used A- and X-site ions within lead-based perovskites. This broadens the applicability of this study to a wider range of compositions that contain the same components. We show that the ability of these additives to alter the stoichiometry of the perovskite bulk leads to significant reduction of device performance. Subsequently, we combine the additives with additional FAI to restore the stoichiometry to the precise ABX_3_ composition and demonstrate that this leads to a full recovery of device performance. The amount of FAI that is required to restore the stoichiometry depends on whether the additive is built into the perovskite lattice. Hence, for PbCl_2_ one equivalent of FAI is needed because chloride is built into the perovskite lattice, but for Pb­(SCN)_2_ three equivalents of FAI are required because thiocyanate is not incorporated into the perovskite. To support these results, we show that disturbing the stoichiometry of an additive-free perovskite absorber results in similar changes in performance. We conclude that maintaining the bulk stoichiometry of perovskites during material optimization is essential for understanding the intrinsic effects of lead-based additives on the performance of perovskite solar cells.

## 2 Experimental Section

### 2.1 Materials

Unless mentioned otherwise, all materials were used as received without purification and stored under an inert atmosphere. Lead bromide (PbBr_2_, >98%), lead iodide (PbI_2_, 99.99%, trace metal basis), lead chloride (PbCl_2_, >99%), lead thiocyanate (Pb­(SCN)_2_, >98%), [4-(3,6-dimethyl-9H-carbazol-9-yl)­butyl]­phosphonic acid (Me-4PACz, >98%), and 1,6-hexylene diphosphonic acid (HDPA, >98%) were purchased from TCI chemicals. Formamidinium iodide (FAI, >99.99%) and propane-1,3-diammonium iodide (PDAI_2_) were purchased from Greatcell Solar Materials. Cesium iodide beads (CsI, 99.999%) and aluminum oxide nanoparticles (Al_2_O_3_, 20 wt % in IPA) were purchased from Sigma-Aldrich. Phenyl-C_61_-butyric acid methyl ester (PCBM, 99%) was purchased from Solenne BV. Nickel oxide nanoparticle ink (NiO_*x*_, 2.5 wt % in ethanol) was purchased from Avantama. Bathocuproine (BCP, >99.5%) was purchased from Lumtec. Dimethylformamide (DMF, anhydrous 99.9%), dimethyl sulfoxide (DMSO, anhydrous 99.9%), anisole (anhydrous 99.7%), propan-2-ol (IPA, anhydrous, 99.95%), and chlorobenzene (CB, anhydrous 99.8%) were purchased from Sigma-Aldrich. Ethanol (EtOH, > anhydrous 95%) and dodecyl sodium sulfate (99%) were purchased from Acros Organics.

### 2.2 Solution Preparation

All solutions were prepared and spin coated in a N_2_-filled glovebox. A stoichiometric Cs_0.2_FA_0.8_Pb­(I_0.6_Br_0.4_)_3_ perovskite solution (1.2 M) was prepared by dissolving PbI_2_ (221.3 mg, 0.48 mmol), PbBr_2_ (264.3 mg, 0.72 mmol), FAI (165.1 mg, 0.96 mmol) and CsI (62.4 mg, 0.24 mmol) in 1 mL of DMF/DMSO 4:1 (v/v). For additive-containing solutions, 0.5–2 mol % of PbCl_2_ or Pb­(SCN)_2_ was added to the precursor solution, alongside with 0–4 mol % of FAI. For nonstoichiometric precursors, 1–4 mol % of either FAI or PbI_2_ was added to or removed from the precursor. The precursor solution was stirred at 60 °C for 60 min and cooled to room temperature prior to deposition. The NiO_*x*_ nanoparticle dispersion was prepared by diluting the NiO_*x*_ nanoparticle ink with EtOH at a ratio of NiO_*x*_/EtOH 1:10 (v/v). Me-4PACz was dissolved in EtOH (0.5 mg mL^–1^). The commercial Al_2_O_3_ nanoparticle dispersion (20 wt % in IPA) was diluted with IPA in a 1:150 (v/v) ratio. For top passivation, PDAI_2_ was dissolved in a mixture of IPA/CB 2:1 (v/v) (0.5 mg mL^–1^) with stirring at 60 °C for 1 h. PCBM was dissolved in CB (20 mg mL^–1^) and stirred at 60 °C for 1 h. HDPA was dissolved in EtOH (0.3 mg mL^–1^).

### 2.3 Solar Cell Fabrication

Prepatterned indium tin oxide (ITO) (active areas of 0.09 and 0.16 cm^2^) glass substrates (Naranjo, 15 Ω sq^–1^) were sequentially cleaned by sonication in acetone for 15 min, scrubbing and 15 min of sonication in an aqueous sodium dodecyl sulfate solution, rinsing with deionized water for 15 min, and sonication in 2-propanol for 15 min. After drying the substrates with a N_2_ gun, they were treated with ultraviolet (UV)—ozone for 30 min, before being transferring to a N_2_-filled glovebox.

First, 120 μL of NiO_*x*_ was spin coated at 3000 rpm with 1000 s^–1^ acceleration for 30 s. Then, 120 μL of Me-4PACz solution was spin coated at 3000 rpm with 1000 rpm s^–1^ acceleration for 30 s, after which these layers were annealed together for 10 min at 100 °C. After cooling down to room temperature, 120 μL of Al_2_O_3_ dispersion was spin coated at 4000 rpm with 2000 rpm s^–1^ acceleration for 30 s and annealed at 100 °C for 5 min. For the perovskite, 120 μL of precursor solution was spin coated at 4000 rpm with 1000 rpm s^–1^ acceleration for 32 s, and 150 μL anisole was dropped after 28 s from the start of spinning. The perovskite layer was then annealed for 15 min at 100 °C. After cooling to room temperature, 120 μL of PDAI_2_ solution was dynamically spin coated at 4000 rpm for 30 s and annealed at 100 °C for 5 min. Then, 120 μL of PCBM solution was spin coated at 1000 rpm with 1000 rpm s^–1^ acceleration for 30 s, without further annealing. The samples were then transferred to a thermal evaporator, where 8 nm of BCP and 100 nm of Ag were deposited under high vacuum (<10^–7^ Torr).

### 2.4 Film Characterization

Scanning electron microscopy (SEM) images were collected with a FEI Quanta 3D FEG microscope (5 keV electron beam, secondary electron detector) and a PhenomProX (5 keV electron beam, secondary electron detector). X-ray diffraction (XRD) was recorded with a Bruker 2D phaser (Cu Kα radiation, λ = 1.5406 Å). Measurements were performed in the range of 10–40° with a step size of 0.05° and a collection time of 0.5 s. A divergence slit of 0.6 mm and an antiscatter screen of 0.5 mm were used. X-ray photoelectron spectroscopy (XPS) measurements were performed using a Thermo-Scientific K-Alpha with a 180° double-focusing hemispherical analyzer and a 128-channel detector. Monochromatic Al Kα (1486.6 eV) radiation was used, and the X-ray spot size was 400 μm. The depth-profile measurements were performed in etching mode with an ion energy of 500 eV and low current (sputter rate estimate of 0.05 nm s^–1^). Each etch cycle had a duration of 30 s and 90 total levels were measured.

### 2.5 Device Characterization

Solar cells were tested in a N_2_-filled glovebox at ambient temperature. To emulate approximately 100 mW cm^–2^ AM1.5G light, a tungsten halogen lamp in combination with a Schott GG385 UV filter, and Hoya LB120 daylight filter were used. Incident light was referenced using a Si photodiode. Shadow masks of 0.0676 or 0.1296 cm^2^ were used to define the illuminated area of the solar cell. Current density—voltage (*J*–*V*) characteristics were determined using a Keithley 2400 SMU. The *J*–*V* scan swept the applied voltage bias (without prebiasing) from +1.5 to −0.1 V for a reverse scan, or from −0.1 to +1.5 V for a forward scan using a scan rate of 250 mV s^–1^ with 161 steps. For regular external quantum efficiency (EQE) measurements, a tungsten halogen lamp (Philips Focusline, 50 W) was used and its light was mechanically chopped at 165 Hz (Stanford Research SR540) before passing through a monochromator (Oriel Cornerstone 130) and an aperture (0.0314 cm^2^). The cell response was measured using a low-noise current preamplifier (Stanford Research SR570) in combination with a lock-in amplifier (Stanford Research SR830). The incident light intensity was referenced using a Si detector. 1-Sun light bias was simulated using a 530 nm LED (Thorlabs M530L3) driven by a Thorlabs DC4104 driver to accurately determine short-circuit current density (*J*_sc_) under approximately AM1.5G conditions. Highly sensitive EQE measurements used the light from an Osram 64655 HLX 250 W tungsten halogen lamp mechanically chopped at 333 Hz passing appropriate sorting filters and dispersed using an Oriel Cornerstone 260 monochromator. The response was recorded using a Stanford Research SR570 preamplifier and a Stanford Research SR830 lock-in amplifier. Calibration was performed using reference Si and InGaAs detectors. The measured highly sensitive EQE spectra were scaled to regular EQE data.

### 2.6 Quasi-Fermi Level Splitting

The quasi-fermi level splitting (QFLS) was assessed through steady-state absolute photoluminescence (ss-PL) measurements. Samples were excited using a 455 nm Thorlabs M455F3 fiber-coupled LED. Samples were placed under an Avantes AvaSphere-30-REFL integrating sphere equipped with in-line filter holders for excitation light and emitted light, holding a 550 nm short-pass filter (Edmund Optics) and a 550 nm long-pass filter (Edmund Optics), respectively. The incident photon flux was adjusted to simulate AM1.5G conditions. The integrating sphere was connected to an Avantes AvaSpec-HSC1024 × 58TEC-EVO spectrometer (550–1100 nm) by an optical fiber. The setup was calibrated using an Avantes halogen lamp, yielding a spectral correction factor. Spectral photon fluxes ϕ_PL_ were obtained after a Jacobian transformation. Using the nonlinear least-squares fit method in MATLAB, the QFLS (Δ*E*_F_) was determined from the ϕ_PL_. The relation between QFLS and photon flux is defined as follows

$$\phi_{PL}\left(E\right)=\frac{1}{4\pi^{2}h^{3}c^{2}}\frac{a\left(E\right)E^{2}}{\exp\left(\frac{E-\Delta E_{F}}{k_{b}T}\right)-1}$$

where a­(*E*) is the photon energy-dependent absorptivity, which is assumed to be unity for photon energies sufficiently larger than the optical bandgap. Each film or (partial) stack combination was measured on 3 spots on the same film and on multiple films, the QFLS values were averaged and the standard deviation was determined. Next to variations in QFLS for different spots on the sample, there are batch-to-batch variations to inevitable small differences in the composition of the precursor solutions and processing conditions during the investigations. From the available data the standard deviation between nominally identical samples is estimated to be 15 meV or less.

## 3 Results and Discussion

### 3.1 Lead Thiocyanate and Lead Chloride Additives

To study the effect of lead-salt additives, we fabricate inverted (*p*–*i*–*n*) ITO|NiO_*x*_|Me-4PACz|Al_2_O_3_|Cs_0.2_FA_0.8_Pb­(I_0.6_Br_0.4_)_3_|PDAI_2_|PCBM|BCP|Ag solar cells. The Cs_0.2_FA_0.8_Pb­(I_0.6_Br_0.4_)_3_ perovskite layer is processed from a single precursor solution, using anisole as antisolvent, and thermal annealing to create a polycrystalline perovskite thin film. In optimized devices, this wide-bandgap perovskite (1.77 eV) provides a *V*_oc_ of 1.30 V, a *J*_sc_ of 16.5 mA cm^–2^, a fill factor (FF) of 0.82, and a PCE of 17.5% (Figure S1). Adding Pb­(SCN)_2_ into the precursor solution in molar ratios in the range of 0.5–2 mol % with respect to lead, results in a decrease of all photovoltaic parameters (Figures 1 and S1). We attribute this loss in performance primarily to a disturbance of the perovskite stoichiometry. It is widely reported that SCN^–^ anions volatize during thermal annealing of the perovskite layer, according to the following reactions^19^

1 $$2{\left({NH}_{2}\right)}_{2}CHI+{Pb\left(SCN\right)}_{2}\to {PbI}_{2}+2{\left({NH}_{2}\right)}_{2}CHSCN$$

2 $${\left({NH}_{2}\right)}_{2}CHSCN\to {NH}_{2}CHNH↑+HSCN↑$$

**Figure 1:**
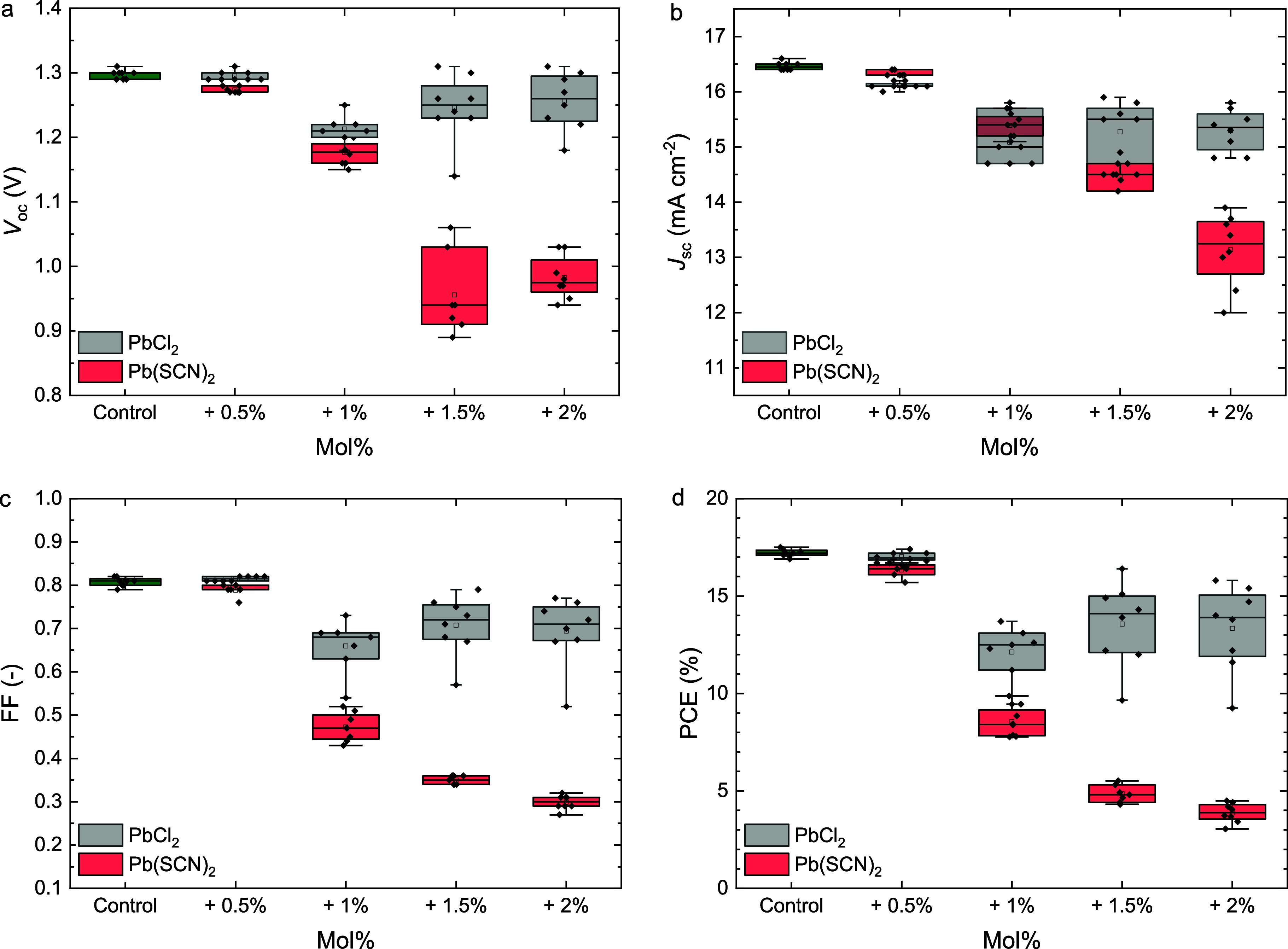
Boxplots of the photovoltaic parameters (a) *V*_oc_, (b) *J*_sc_, (c) FF, and (d) PCE, of ITO|NiO_*x*_|Me-4PACz|Al_2_O_3_|Cs_0.2_FA_0.8_Pb­(I_0.6_Br_0.4_)_3_|PDAI_2_|PCBM|BCP|Ag solar cells processed without and with different mol % of Pb­(SCN)_2_ or PbCl_2_ as additive to the precursor solution. The boxplots show the mean (open square), median (center line), 25th and 75th percentiles (box limits), and minimum and maximum (whiskers).

These reactions imply that the addition of Pb­(SCN)_2_ results in the formation of PbI_2_ and a deficiency of FAI, because a proton of FAI is consumed to form the volatile thiocyanic acid (HSCN), which leads to the concomitant loss of formamidine (NH_2_CHNH). The formation of PbI_2_ is evidenced by the appearance of the characteristic (001) reflection at 12.7° in the XRD (Figure 2a) and by the formation of PbI_2_ crystallites on the perovskite surface in the SEM images (Figure S2). We note that the intensities of the Bragg peaks varied somewhat between nominally identical samples and should not be interpreted quantitatively; they rather serve as a qualitative description of the structural composition of the films. XPS surface scans of films processed with 5 mol % of Pb­(SCN)_2_ (Figure S3) confirm the formation of excess PbI_2_ by the increased intensity of the I 3d and Pb 4f photoelectron peaks, implying higher I^–^ and Pb^2+^ contents, and a I^–^/Pb^2+^ ratio closer to 2 (2.73 without additive, and 2.61 with additive) at the top surface of films processed with 5 mol % Pb­(SCN)_2_.

**Figure 2:**
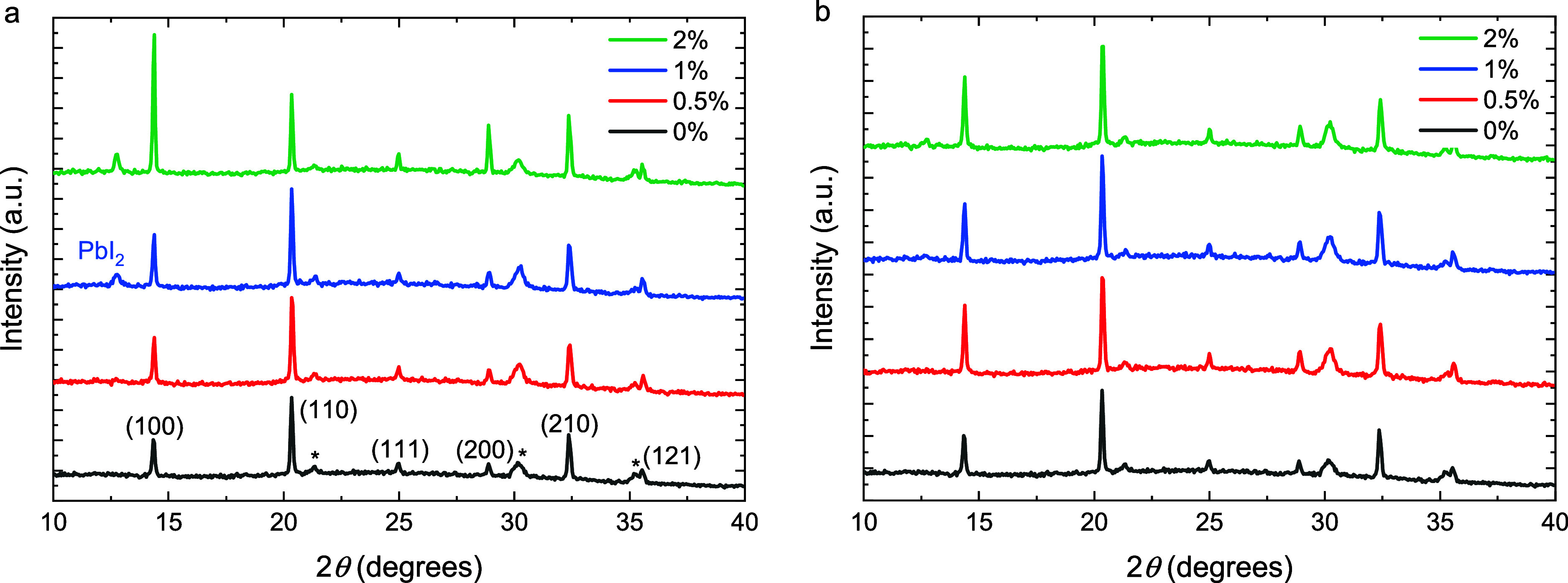
X-ray diffractograms of stoichiometric Cs_0.2_FA_0.8_Pb­(I_0.6_Br_0.4_)_3_ perovskite films (0 mol %) and films processed with 0.5, 1, and 2 mol % of additive. (a) Pb­(SCN)_2_. (b) PbCl_2_. Peaks were assigned by assuming a cubic unit cell in the space group *Pm*3̅*m*. Peaks indicated with an asterisk are from ITO.

These results demonstrate that the annealed Cs_0.2_FA_0.8_Pb­(I_0.6_Br_0.4_)_3_ films do not have the overall perfect ABX_3_ stoichiometry when processed with Pb­(SCN)_2_ as an additive, but rather exist in a form with deficiencies of the A-site (FA^+^, Cs^+^) and X-site (I^–^, Br^–^) ions and thus an excess of B-site (Pb^2+^) cations. Tauc plots reveal that increasing the concentration of the Pb­(SCN)_2_ additive widens the bandgap by up to approximately 7 meV at 2 mol % (Figure S4). Qualitatively, the wider bandgap is explained by the evaporation of formamidine from the Cs_0.2_FA_0.8_Pb­(I_0.6_Br_0.4_)_3_ perovskite film which increases the Cs^+^/FA^+^ ratio, and by the formation of PbI_2_, which increases the Br^–^/I^–^ ratio within the perovskite phase. Assuming that reactions (1) and (2) occur to the full extent, the addition of α mole of Pb­(SCN)_2_ per mole of lead results in a change of perovskite composition given by

3 $$0.4{PbI}_{2}+0.6{PbBr}_{2}+0.8FAI+0.2CsI+\alpha {Pb\left(SCN\right)}_{2}\to 2\alpha FASCN\left(↑\right)+3\alpha {PbI}_{2}+\left(1-2\alpha \right){Cs}_{0.2/\left(1-2\alpha \right)}{FA}_{\left(0.8-2\alpha \right)/\left(1-2\alpha \right)}Pb{\left[I_{\left(0.6-2\alpha \right)/\left(1-2\alpha \right)}{Br}_{0.4/\left(1-2\alpha \right)}\right]}_{3}$$

Hence, at α = 0.02 (2 mol % of additive) the Cs^+^/FA^+^ ratio [1/(4–10α)] changes from the original 0.250 to 0.263 and the Br^–^/I^–^ ratio [1/(1.5–5α)] from 0.667 to 0.714. The slightly higher Cs^+^/FA^+^ ratio would give a rise in bandgap of about 1.5 meV,^26^ whereas the expected increase in bandgap as a consequence of the higher Br^–^/I^–^ is approximately 10 meV, based on experiments where we varied the Br^–^ content of the above-mentioned composition between 0 and 40%. The total expected blue shift of approximately 11.5 meV is larger than the 7 meV shift observed experimentally and indicates a lower Br^–^/I^–^ ratio than 0.714, which can occur when not all 3α PbI_2_ is expelled from the perovskite lattice. This would create a deficiency of A- and X-side ions and a nonstoichiometric perovskite.

The minor A- and X-site ion deficiencies that result from processing with 0.5 mol % of Pb­(SCN)_2_ as additive, reduce device performance only marginally but all parameters are significantly affected at concentrations of 1 mol % or higher (Figure 1). This presumably stems from the significant increase of PbI_2_ at the top surface of the perovskite, which is reported to facilitate charge recombination and thus reduce the *V*_oc_,^27^ while simultaneously hampering charge extraction due to its insulating nature.^28^

When PbCl_2_ is used as an additive, the Cs_0.2_FA_0.8_Pb­(I_0.6_Br_0.4_)_3_ solar cells show a much smaller drop in performance (Figure 1). A small amount (0.5 mol %) of PbCl_2_ even slightly improves the *V*_oc_ by 10 mV, in agreement with previous studies where PbCl_2_ was found to facilitate the incorporation of Cl^–^ into the perovskite lattice, resulting in the passivation of trap states and an increased charge mobility, which both reduce charge recombination losses.^29^ However, the small increase in *V*_oc_ might also stem from the slight increase in bandgap due to the substitution of iodine with chloride. For this concentration, the slight increase in *V*_oc_ is accompanied by a change in the XRD pattern (Figure 2b) where the intensity of the (100) diffraction peak at 14.4° increases compared to the (200) peak at 28.8° without an observable shift of the (100) peak position. Further increasing the PbCl_2_ concentration in the precursor solution leads to a reduction of *J*_sc_, which is again ascribed to the formation of excess lead iodide at the surface that acts as an insulating layer, as shown in the SEM images (Figure S5).^28^ It is evident that a deviation from the ideal stoichiometry is detrimental for device performance.

Depth profiling XPS (Figures 3 and S6) on films with 5 mol % of additive reveals that chlorine is present in the annealed perovskite films processed with PbCl_2_, but that—in contrast—no sulfur is present when Pb­(SCN)_2_ was used. Apparently Cl^–^, with its smaller ionic radius than Br^–^ or I^–^, is easily built into the perovskite lattice but SCN^–^ is not. Although the radius of SCN^–^ (2.13 Å) has been reported to be in between that of Br^–^ (1.96 Å) and I^–^ (2.20 Å),^30^ the length of approximately 3 Å of the linear SCN^–^ ion makes that it cannot be accommodated in the regular cubic perovskite crystal structure. Accordingly, A_2_Pb­(SCN)_2_X_2_ perovskites (with A = MA, FA, or Cs and X = Br or I) adopt a layered structure in which Pb^2+^ is octahedrally coordinated by four halide ions and by two S-bonded thiocyanate ligands in a trans position with their N-termini directed to the interlayer space.^31–34^ Thus, the favorable Pb–S bonding compared to Pb–N bonding, causes that Pb­(SCN)_2_X_4_ octahedra are linked into layers through corner-sharing halide ligands, while the thiocyanate ligands disconnect the octahedral network. Incorporation of thiocyanate into a FAPbI_3_ perovskite to form FA_6_Pb_4_I_13·5_(SCN)_0.5_ and FA_4_Pb_2_I_7·5_(SCN)_0.5_ phases has been shown to result in columnar defects.^35,36^ When using Pb­(SCN)_2_ as an additive in small molar excess, such columnar defects are likely to form at grain boundaries and emerge at the crystallite surfaces.

**Figure 3:**
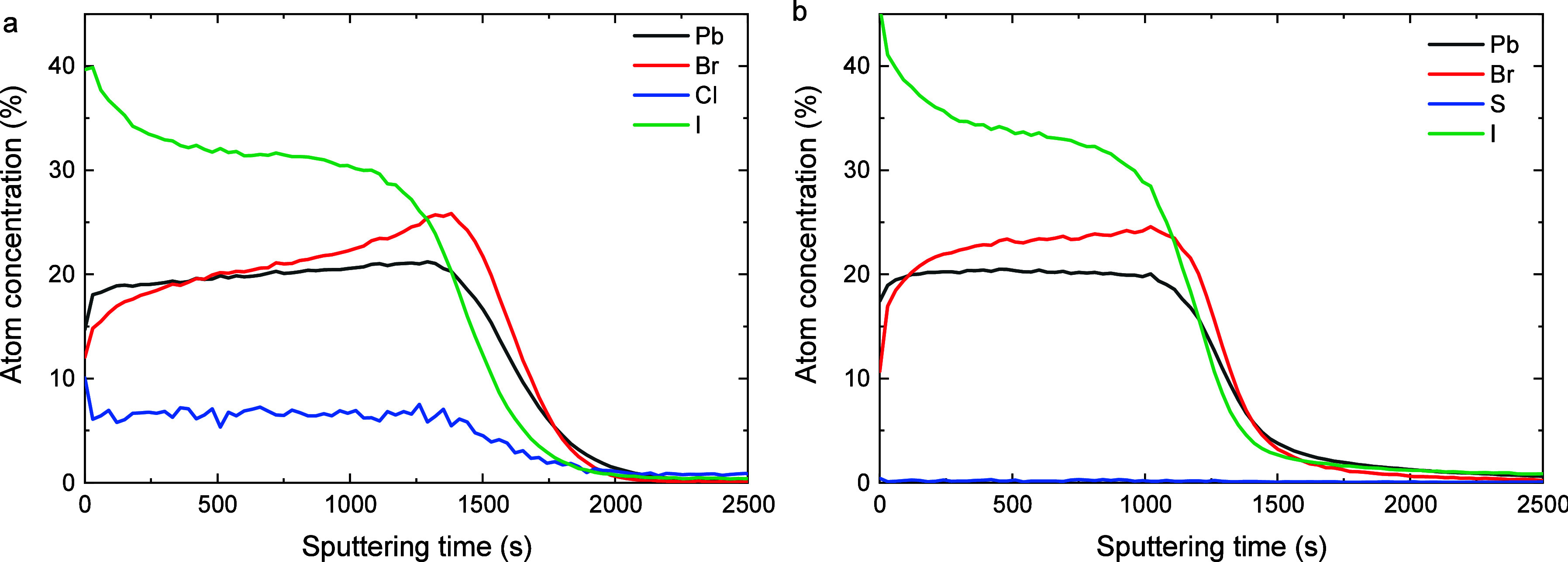
XPS-depth profiles of a perovskite alloyed with (a) 5 mol % PbCl_2_ and (b) 5 mol % of Pb­(SCN)_2_. More detailed information regarding the elemental distribution is shown in Figure S6.

When PbCl_2_ is converted into perovskite, it consumes one equivalent of FAI to maintain stoichiometry, according to

4 $${\mathrm{PbCl}}_{2}+{{\left({NH}_{2}\right)}_{2}CHI\to \left[{\left({NH}_{2}\right)}_{2}CH\right]Pb\left({Cl}_{0.66}I_{0.33}\right)}_{3}$$

and assuming that α mole PbCl_2_ as additive is incorporated into the perovskite, this would leave α mole of PbI_2_ unreacted, according to

5 $$0.4{PbI}_{2}+0.6{PbBr}_{2}+0.8FAI+0.2CsI+\alpha {PbCl}_{2}\to \alpha {PbI}_{2}+{Cs}_{0.2}{FA}_{0.8}Pb{\left[I_{\left(0.6-2\alpha \right)/3}{Br}_{0.4}{Cl}_{2\alpha /3}\right]}_{3}$$

In contrast to the perovskite films deposited with Pb­(SCN)_2_, the use of PbCl_2_ does not change the initial Cs^+^/FA^+^ ratio of 0.25. When 2 mol % (α = 0.02) of PbCl_2_ is used as additive, the Br^–^/I^–^ ratio [1/(1.5–5α/3)] changes from 0.667 to 0.682, which is expected to blue-shift the bandgap by approximately 3 meV. Additionally, the Cl^–^/I^–^ ratio [2α/(1.8–2α)] changes from 0 to 0.023, resulting in an estimated blue-shift of the bandgap by approximately 11 meV.^37^ Again, the total expected shift of 14 meV is somewhat larger than the experimental value of approximately 7 meV (Figure S7).

As noted, the observed bandgap shifts of 7 meV when using 2 mol % Pb­(SCN)_2_ or PbCl_2_ are slightly smaller than the shifts of 11.5 and 14 meV, predicted when assuming that reactions (3) and (5) occur to the full extent. The uncertainty of the experiment and the predictions is several meV, which may explain this difference in part. It is also possible that the lead halide expelled in reactions (3) and (5) is not exclusively PbI_2_, but also contains PbBr_2_ or PbCl_2_. In such case the resulting Br^–^/I^–^ ratio is closer to the original value of 0.667. This would reduce the difference between the expected and experimentally obtained shifts. However, XPS depth profiling (Figures 3a and S6) suggests an increased concentration of iodide, and possibly chloride, at the surface, suggesting that mainly PbI_2_ is formed. We have found no evidence for the formation of other phases from XRD. In case quasi-2D domains would form,^35,36^ these would have a much wider bandgap.

### 3.2 Compensating for the Loss of Stoichiometry

Following reaction (4), the addition of PbCl_2_ consumes one equivalent of FAI when it is converted into perovskite and leaves one equivalent of lead halide unreacted. In contrast, the combination of reactions (1) and (2) shows that addition of Pb­(SCN)_2_ creates a 3-fold deficiency in FAI: two FAI equivalents are necessary to compensate for the evaporation of two formamidine molecules and one FAI to convert the PbI_2_ that is formed in reaction (1) into a perovskite. To confirm that disturbing the ABX_3_ stoichiometry is the primary cause for degrading device performance, we employed either Pb­(SCN)_2_ or PbCl_2_, together with additional FAI to restore the ABX_3_ stoichiometry by supplying additional A-site and X-site ions to react with the excess Pb^2+^ stemming from the lead-salt additives. Following this concept, the equivalents of FAI required to restore the stoichiometry and device performance should be related to the fraction of the lead-salt additive that is built into the perovskite lattice. This implies that, according to reaction (4), adding PbCl_2_ to the perovskite precursor requires one additional equivalent of FAI to be added to ensure full conversion of the additives to a stoichiometric photoactive phase. On the other hand, the absence of SCN^–^ in the perovskite indicates that Pb­(SCN)_2_ merely provides excess Pb^2+^ to the annealed additive-rich film, while consuming the available FAI to form formamidine and thiocyanic acid, according to reactions (1) and (2). Accordingly, three additional equivalents of FAI are required per equivalent of Pb­(SCN)_2_ added to achieve full conversion of the additive into a stoichiometric photoactive perovskite phase, according to

6 $$3{\left({NH}_{2}\right)}_{2}CHI+{Pb\left(SCN\right)}_{2}\to {\left[{\left({NH}_{2}\right)}_{2}CH\right]PbI}_{3}+2{\left({NH}_{2}\right)}_{2}CHSCN\left(↑\right)$$

there with effectively adding [(NH_2_)_2_CH]­PbI_3_ to the perovskite composition.

To test our hypothesis, we added 1 mol % of PbCl_2_ or Pb­(SCN)_2_ into the perovskite precursor solution, along with 1–4 mol % of excess FAI. As illustrated in Figure 4, the photovoltaic parameters show that employing 1 mol % of PbCl_2_ or Pb­(SCN)_2_ as additive restores device performance when the addition is compensated by a specific additional amount of FAI, which is one equivalent of FAI for PbCl_2_ and three equivalents of FAI for Pb­(SCN)_2_. Adding α mole of PbCl_2_ and α mole of FAI per mole of lead to the precursor solution leads to a perovskite composition given by

7 $$0.4{PbI}_{2}+0.6{PbBr}_{2}+\left(0.8+\alpha \right)FAI+0.2CsI+\alpha {PbCl}_{2}\to \left(1+\alpha \right){Cs}_{0.2/\left(1+\alpha \right)}{FA}_{(0.8+\alpha )/\left(1+\alpha \right)}Pb{\left[I_{(0.6+0.33\alpha )/\left(1+\alpha \right)}{Br}_{0.4/\left(1+\alpha \right)}{Cl}_{0.66\alpha /(1+\alpha )}\right]}_{3}$$

whereas adding α mole of Pb­(SCN)_2_ and 3α mole of FAI per mole of lead to the precursor leads to

8 $$0.4{PbI}_{2}+0.6{PbBr}_{2}+\left(0.8+3\alpha \right)FAI+0.2CsI+\alpha {Pb\left(SCN\right)}_{2}\to 2\alpha FASCN\left(↑\right)+\left(1+\alpha \right){Cs}_{0.2/\left(1+\alpha \right)}{FA}_{(0.8+\alpha )/\left(1+\alpha \right)}Pb{\left[I_{(0.6+\alpha )/\left(1+\alpha \right)}{Br}_{0.4/\left(1+\alpha \right)}\right]}_{3}$$

**Figure 4:**
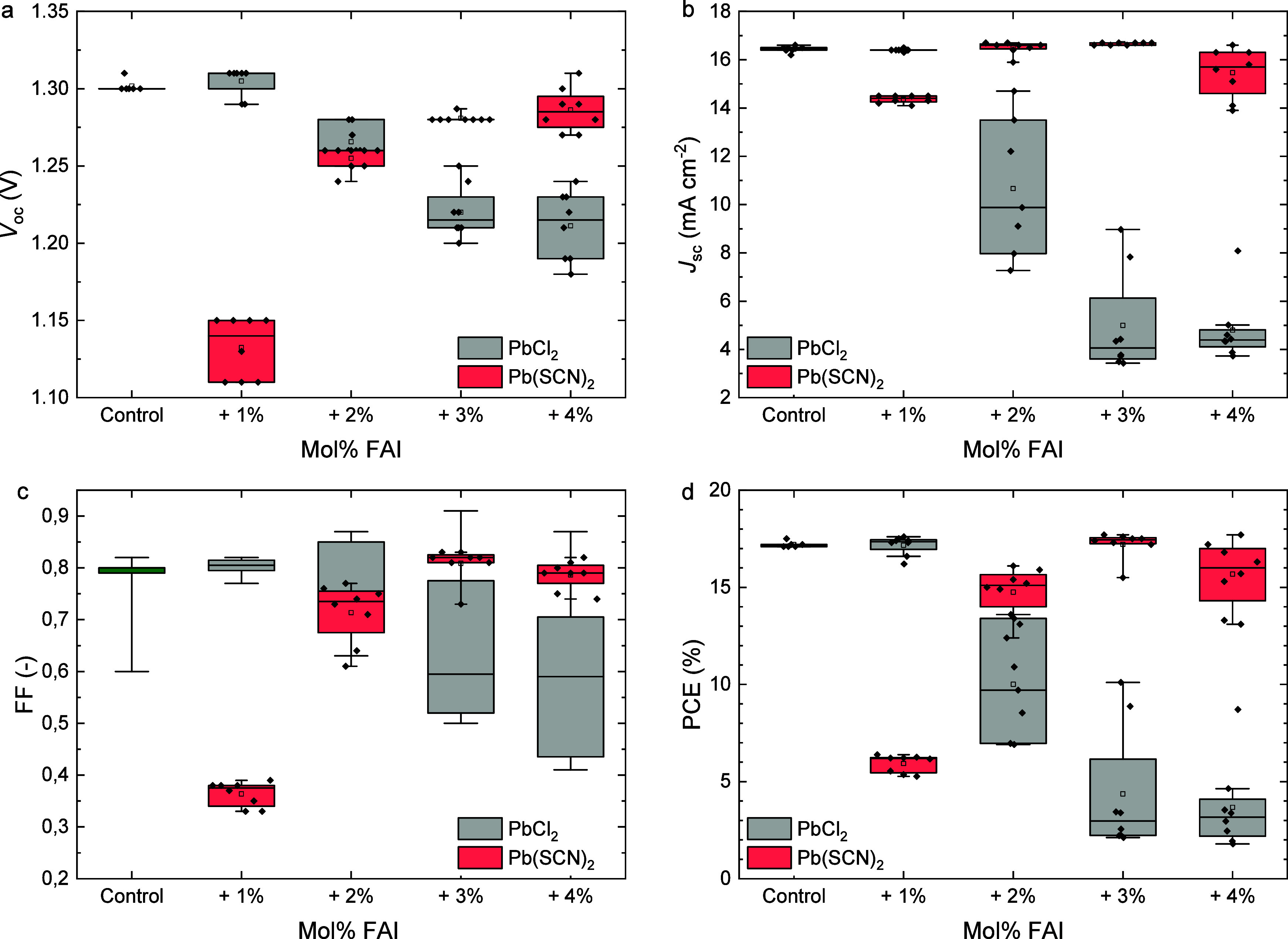
Boxplots of the photovoltaic parameters (a) *V*_oc_, (b) *J*_sc_, (c) FF, and (d) PCE, of ITO|NiO_*x*_|Me-4PACz|Al_2_O_3_|Cs_0.2_FA_0.8_Pb­(I_0.6_Br_0.4_)_3_|PDAI_2_|PCBM|BCP|Ag solar cells (8 devices per variation, measured as reverse scan) processed without and with 1 mol % Pb­(SCN)_2_ or PbCl_2_ as additive to the precursor solution together with 1–4 mol % excess FAI. The boxplots show the mean (open square), median (center line), 25th and 75th percentiles (box limits), and minimum and maximum (whiskers).

The difference between PbCl_2_ and Pb­(SCN)_2_ supports the idea that Cl^–^ does not volatize as HCl during thermal annealing. In that case, the overall stoichiometry would not be maintained in the annealed film with a 1:1 molar ratio of PbCl_2_:FAI, and one equivalent of FAI would still result in an X-site anion deficiency. However, Figure 4 shows that exceeding the required 1:1 ratio of FAI: PbCl_2_ is detrimental to device performance, especially regarding the *J*_sc_. This observation is already widely reported in the literature and stems from the accumulation of excessive organic cations due to ion migration at the interface that severely hampers charge extraction, since this inevitably leads to energy level misalignment and increased recombination losses.^38^ This is also recognized by the significant increase in hysteresis when adding additives in increasing amounts (Figure S8a). X-ray diffractograms (Figure S9b) reveal the presence of a small amount of PbI_2_ in the reference sample and in the sample with (1 mol % PbCl_2_ + 1 mol % FAI), which is supported by SEM images (Figure S10) that show white crystallites on top of the perovskite surface. No PbI_2_ is found once the FAI excess exceeds 1 mol %, confirming that excess FAI binds to excess PbI_2_ that would otherwise reside on the perovskite surface. Increasing the FAI excess beyond 1 mol % increases the (100)/(110) peak ratio, but also leads to the formation of pinholes as shown in Figure S10, possibly due to the larger excess of volatile FAI. Finally, Tauc plots (Figure S11d) show a blue-shift of the bandgap with 1 mol % of PbCl_2_, as was shown previously. Simultaneous addition of 1 or 2 mol % of excess FAI shifts the bandgap toward that of the reference, whereas a significant red-shift of the bandgap is observed when adding more excess FAI (3 4 mol %). This suggests that 1 mol % of FAI leads to a stoichiometric perovskite when 1 mol % of PbCl_2_ is used as additive.

For Pb­(SCN)_2_, a FAI deficiency severely affects both the *V*_oc_ and FF, while the *J*_sc_ remains somewhat unaffected, as shown in Figure 4. The large drop in voltage and FF is expected due to the presence of halide vacancies stemming from the FAI deficiency, as well as the unformed PbI_6_^4–^ octahedra due to a deficiency of both A- and X-site ions. The ideal 1:3 ratio of Pb­(SCN)_2_:FAI supports our assumption that SCN^–^ is fully volatized during annealing and is not built into the lattice. Hence, according to reaction (4), the consumed FAI must be compensated for, while the excessively formed PbI_2_ requires additional FAI to be converted into the photoactive FAPbI_3_ phase. This reaction mechanism is further verified by X-ray diffractograms (Figure S9a), where a FAI excess below 2 mol % leads to a significant trace of residual PbI_2_. SEM images (Figure S10) further verify that 3 mol % of excess FAI is required to eliminate undesirable PbI_2_ crystals on the perovskite surface. Similarly to using PbCl_2_, providing more FAI than required to compensate for the disturbed stoichiometry increases the (100)/(110) peak ratio, but has negative effects on device performance due to a combination of pinholes and the presence of excessive organic cations. Tauc plots (Figure S11c) show a blue-shift of the bandgap when using Pb­(SCN)_2_ and reveal only minor shifts for FAI excesses below 3 mol %. Adding 3 mol % of excess FAI red-shifts the bandgap slightly beyond that of the reference, but a significant red-shift is observed at 4 mol % which exceeds the FAI excess that is required to restore the stoichiometry. This illustrates that beyond the optimal FAI excess, the iodide/bromide ratio within the absorber is affected.

Both the depth-profile XPS (Figure 3) and the blue-shift of the bandgap with increasing PbCl_2_ concentration (Figure S7) support that Cl^–^ is built into the lattice while SCN^–^ is not. The results shown in Figure 4 demonstrate that adding additional FAI in the correct amount can compensate for the offset ABX_3_ stoichiometry caused by the lead-salt additives. The different behavior of Cl^–^ and SCN^–^ likely originates from two complementary effects. First, as argued above, Cl^–^ is built easier into the 3D perovskite lattice than SCN^–^ due to their atomic radii. Second, the fact that Cl^–^ is a weaker base than SCN^–^ favors the loss of HSCN (p*K*_a_ ≈ −1) over that of HCl (p*K*_a_ ≈ −6) during thermal annealing.

### 3.3 Perturbing the Precursor Stoichiometry

Having illustrated that maintaining stoichiometry is essential to achieve proper device performance when using lead-salt additives, we delve deeper into the stoichiometry of an additive-free perovskite by deliberately unbalancing the stoichiometry of the optimized precursor solution through solely changing the FAI or PbI_2_ content. Thereby, we intentionally cause an excess or deficiency of either FAI or PbI_2_ within the precursor solution. Figure 5 summarizes the effect on the solar cell performance parameters by deliberately offsetting the stoichiometry by withholding or adding up to 4 mol % of either PbI_2_ or FAI into the precursor solution. The changes in *J*_sc_ for these off-stoichiometry devices seen in Figure 5b are confirmed by the changes in external quantum efficiency (EQE) spectra (Figures S12 and S13).

**Figure 5:**
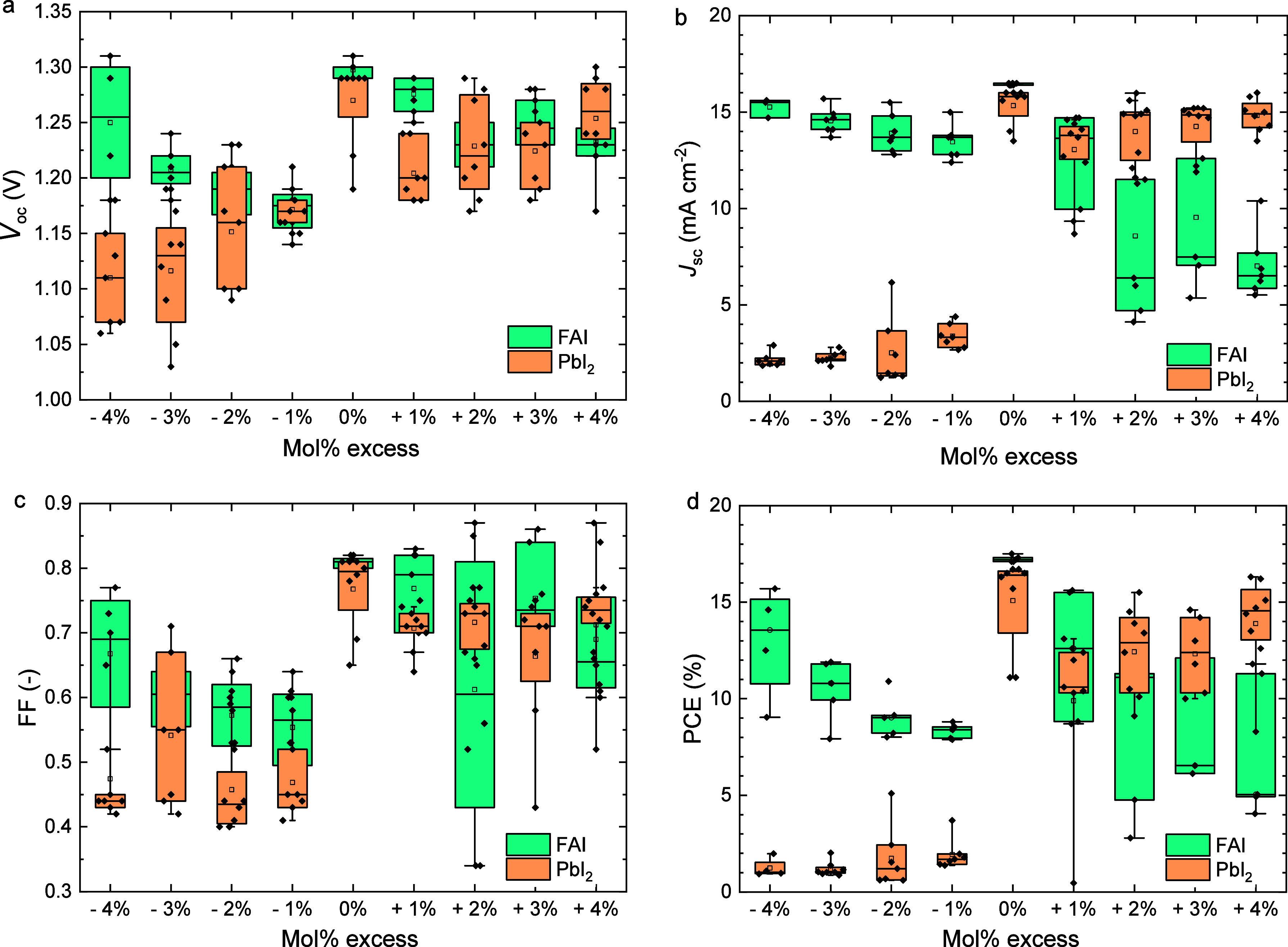
Boxplots of the photovoltaic parameters (a) *V*_oc_, (b) *J*_sc_, (c) FF, and (d) PCE, of ITO|NiO_*x*_|Me-4PACz|Al_2_O_3_|Cs_0.2_FA_0.8_Pb­(I_0.6_Br_0.4_)_3_|PDAI_2_|PCBM|BCP|Ag solar cells (8 devices per variation, measured as reverse scan) processed with −4 to +4 mol % of either PbI_2_ or FAI in the precursor solution. The boxplots show the mean (open square), median (center line), 25th and 75th percentiles (box limits), and minimum and maximum (whiskers). 0% mol excess represents fully stoichiometric films.

Figure 5 reveals that devices made from stoichiometric precursor solutions perform the best and that any deviation from the ideal stoichiometry is detrimental for device performance. Figure 5b shows that a PbI_2_ deficiency leads to a nearly complete loss of photocurrent, which could stem from unformed PbI_6_^4–^ octahedra due to the lack of sufficient Pb^2+^, resulting in an excessive amount of defects that in turn act as charge traps. Likewise, an excess of FAI rapidly reduces the PCE, also mainly by a loss of *J*_sc_, which is likely caused by the accumulation of organic cations near the interface with the electron transport layer (ETL) that impede charge extraction.

On the other hand, a FAI deficiency severely affects the FF and *V*_oc_ due to the presence of both halide vacancies and the deficiency of FA^+^. Surprisingly, the impact of the FAI deficiency and PbI_2_ excess on the PCE lessens when further increasing the offset from the optimum stoichiometry. One speculation for this is that a slight FAI deficiency or PbI_2_ excess does not yet result in the formation of a PbI_2_ phase, but is rather accommodated by creating FA^+^ and I^–^ vacancies in the photoactive perovskite phase. When the FAI deficiency, and thus PbI_2_ excess, becomes sufficiently large, it may form a separate PbI_2_ phase, which then reduces the defect concentration in the perovskite phase. The XRD patterns of the films (Figure 6a) show signatures of PbI_2_ at the highest FAI deficiency. However, it must be noted that aside from the increasing PCE with larger stoichiometric offsets of the FAI deficiency or the PbI_2_ excess, this simultaneously leads to severe hysteresis (Figure S8b), likely stemming from increased ionic movements and rendering these layers unsuitable for solar cells.

**Figure 6:**
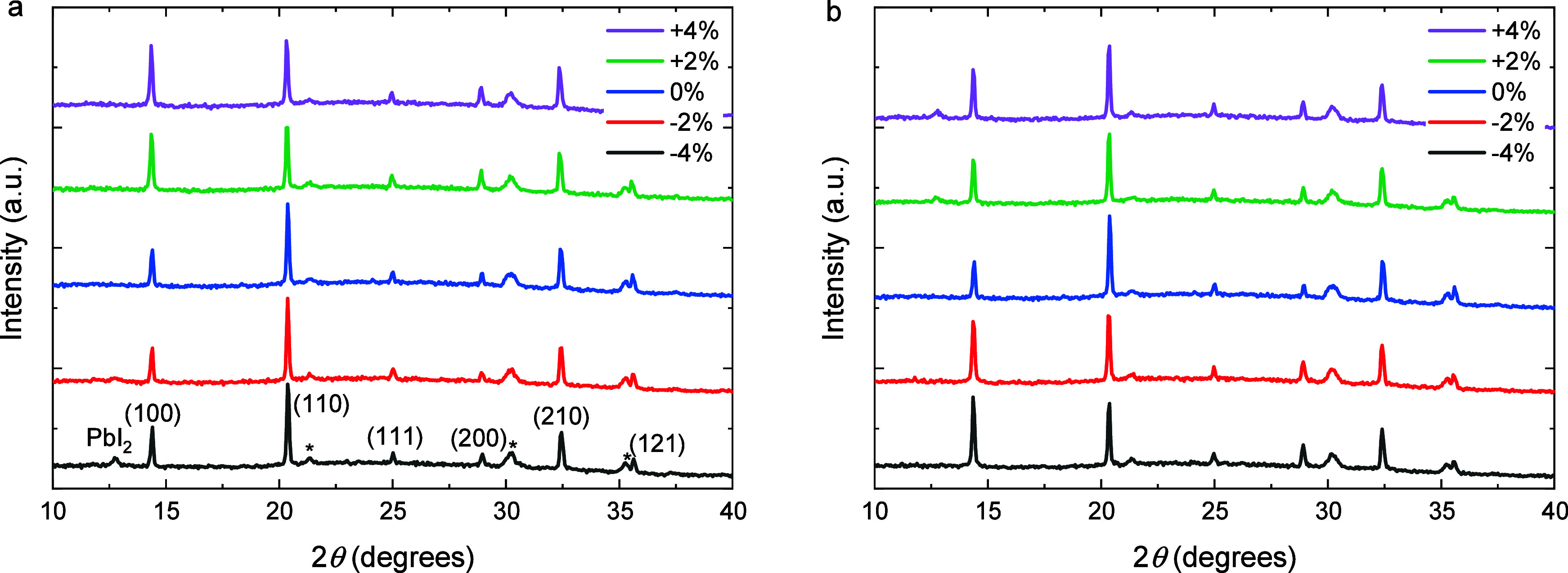
X-ray diffractograms of Cs_0.2_FA_0.8_Pb­(I_0.6_Br_0.4_)_3_ perovskite films prepared from precursor solutions with different mole percent excess or deficiency of either FAI (a) or PbI_2_ (b). Peaks were assigned by assuming a cubic unit cell in the space group *Pm*3̅*m*. Peaks indicated with an asterisk are from ITO.

Figure 6 shows that the changes in the X-ray diffractograms of perovskite films with excess PbI_2_ are similar to those with a deficiency of FAI. With increasing FAI deficiency or PbI_2_ excess, a diffraction peak appears at 12.7°, indicating the formation of PbI_2_, as also observed in the SEM images (Figure S14). Furthermore, the SEM images (Figure S14) verify that increasing the PbI_2_ excess beyond stoichiometric concentration leads to direct expulsion of excess PbI_2_ crystallites toward the perovskite film surface, which obeys a similar mechanism as a deficiency of FAI. On the other hand, an excess of FAI or deficiency of PbI_2_ leads to a significant increase in the number of pinholes in the film and an increasing (100) peak intensity (Figure 6), which is likely related to the increasing excess of FA^+^ that has to be expelled from the film during crystallization of the perovskite layer.

To investigate what happens with these stoichiometric offsets during film fabrication, we measured the absolute photoluminescence (APL) and extracted the QFLS for films with an excess or deficiency of either FAI or PbI_2_. The APL was measured for Cs_0.2_FA_0.8_Pb­(I_0.6_Br_0.4_)_3_ perovskites deposited on glass/ITO substrates functionalized with HDPA. Compared to other substrates and surface treatments, HDPA significantly improved reproducibility and resulted in consistent QFLS values.^39^ Figure S15 shows that only small differences are found when films are measured from either glass or film sides. Surprisingly, any modification to the perovskite composition, albeit an excess or deficiency of either FAI or PbI_2_, leads to a small increase in QFLS (Figure S15), but a PbI_2_ deficiency or a FAI excess seem to have the most impact. Figure S16 shows the normalized PL spectra, which reveal minor variations in peak position (697 ± 3 nm) and peak width (fwhm 44.4 ± 0.8 nm), corresponding to small changes (approximately 15 meV) in bandgap. Even though the improvement of QFLS is small (<40 meV), it does not explain the rapid loss in device performance when the precursor solution deviates from the perfect stoichiometry (Figure 5). This finds its origin in the fact that the optically measured QFLS cannot always be directly correlated to device performance, because it is not sensitive to hindered charge extraction.^40^ Stolterfoht et al. have shown that a mismatch between QFLS and *V*_oc_ is expected to occur when the diffusion of carriers to the metal contact or charge transport layer is slow compared to the nonradiative recombination in the interface region.^41^ Rather, the QFLS provides information on the quality of the perovskite semiconductor and its interfaces with adjacent layers. Note that films with increasing excess of FAI or PbI_2_ show opposite trends, in agreement with the argument that an excess of FAI leads to a deficiency of PbI_2_ and vice versa. Indeed, the QFLS increases with PbI_2_ deficiency or FAI excess, while a PbI_2_ excess or FAI deficiency show minor impact. This suggests that a FAI excess is the main contributor to an increased QFLS. Possibly, the excess FAI migrates toward the top surface of the perovskite and passivates defect states at this interface which increases the QFLS, but at the same time hinders charge collection, resulting in sharp drops in *J*_sc_ and FF (Figure 5).

To verify our assumption and study how the losses at the ETL interface are affected by the absorber stoichiometry, we deposited PCBM on the same films and repeated the APL measurements (Figure 7). Consistent with previous studies,^42^ application of a fullerene-based ETL leads to significant loss of QFLS as a consequence of nonradiative recombination at the perovskite-PCBM interface. The perovskite-PCBM interface is considered the limiting interface to the *V*_oc_, where the degree of the loss is affected by absorber stoichiometry. Again, there is an opposite trend for FAI and PbI_2_. Increasing the FAI excess reduces the interfacial losses, which is also found when increasing the PbI_2_ deficiency. This observation is consistent with the assumption that excess FAI migrates toward the top surface and reduces interfacial losses between the perovskite and PCBM. The advantageous effect of a thin interlayer between the perovskite and fullerene on the QFLS was shown before for choline chloride and LiF.^40,42^

**Figure 7:**
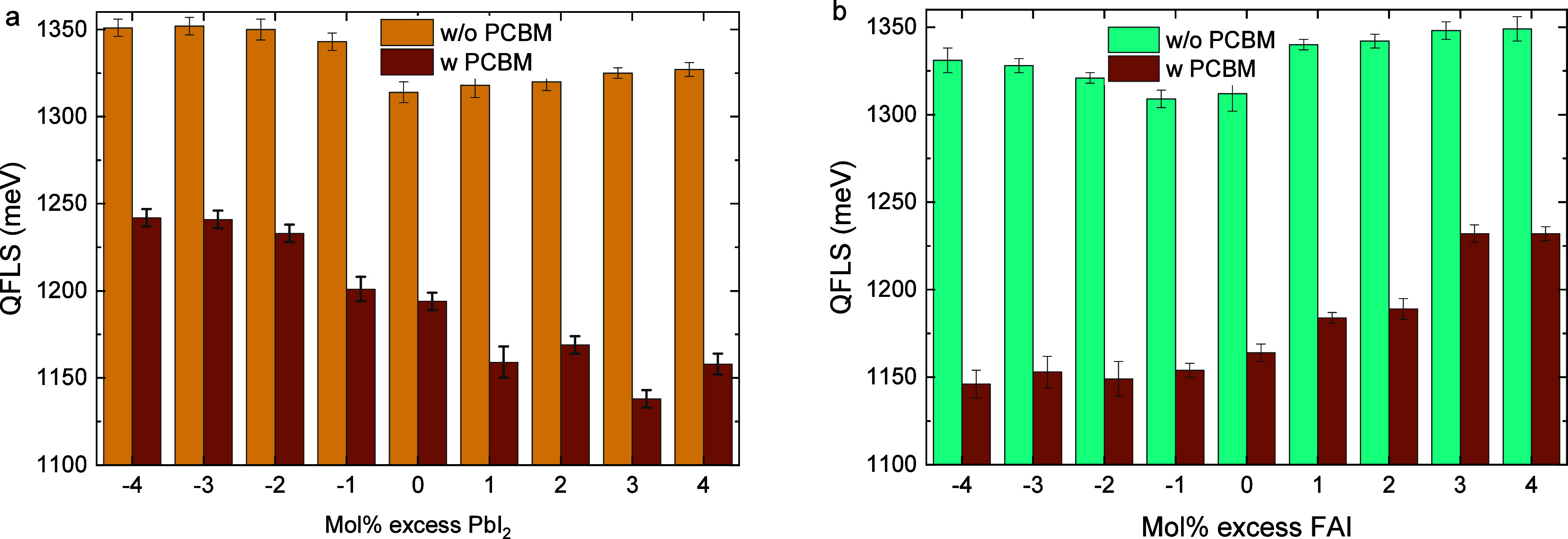
QFLS of Cs_0.2_FA_0.8_Pb­(I_0.6_Br_0.4_)_3_ perovskite films processed from precursor solutions with films with excess or deficiency of PbI_2_ (a) or FAI (b) without and with a PCBM layer on top.

Given the increase in QFLS (Figure 7) and the simultaneous decline in device performance (Figure 5) with any stoichiometric offset, overpassivation—potentially caused by the combined presence of PDAI_2_ and excess FAI or PbI_2_ at the perovskite film surface—cannot be excluded. Therefore, we fabricated devices without interfacial passivation (PDAI_2_), with an excess or deficiency of FAI of up to 4 mol % in the precursor solution, as shown in Figure S17. Once more, we clearly show that the precursor stoichiometry should be conserved to achieve optimal device performance. Any stoichiometric offset leads to a slight reduction of *J*_sc_ by up to 1.5 mA cm^–2^, but its primary impact is on the voltage. With a slight FAI deficiency the *V*_oc_ increases by up to 30 mV, possibly due to the passivating properties of excess PbI_2_ on the perovskite film surface (Figure S14). However, the insulating nature of PbI_2_ and its detrimental effects on the FF and *J*_sc_ result in poor-performing devices. On the other hand, excess FAI leads to a significant loss of *V*_oc_ by approximately 300 mV, likely stemming from increase of perovskite film porosity (Figure S14) due to the volatile FAI or from a FAI-rich surface that hinders charge collection.

Highly sensitive sub-bandgap photocurrent spectra of devices with an excess and deficiency of FAI or PbI_2_ (Figure S18) reveal the presence of two sub-bandgap contributions to the EQE at approximately 0.95 and 1.40 eV that are associated with defects at the perovskite-PCBM interface.^43^ The small variations in peak position are a consequence of small variations in film thickness (420 ± 20 nm) that cause a change of interference of the sub-bandgap light in the layer stack. The exponential band tail allows to determine the Urbach energy (*E*_u_), which is a measure of the energetic disorder in the absorber,^44^ but can also be affected by interfacial defects in the region where the EQE becomes very small. Hence *E*_u_ is not predominantly sensitive to changes in the bulk or at interfaces. Figure 8 shows that *E*_u_ is affected by absorber stoichiometry. This is expected, because a disturbance of the ABX_3_ stoichiometry inevitably results in an increased degree of energetic disorder, and therefore *E*_u_ increases with increasing PbI_2_ deficiency. A PbI_2_ excess, however, has a small effect on the *E*_u_. We propose that excess PbI_2_ is not built into the crystal lattice and apparently has no significant effect on the *E*_u_. When changing the FAI concentration, a low *E*_u_ is found for the stoichiometric composition as expected. For a FAI excess, the *E*_u_ is higher, similar to what is seen for a PbI_2_ deficiency. For a FAI deficiency, the *E*_u_ does not decrease as seen for a PbI_2_ excess.

**Figure 8:**
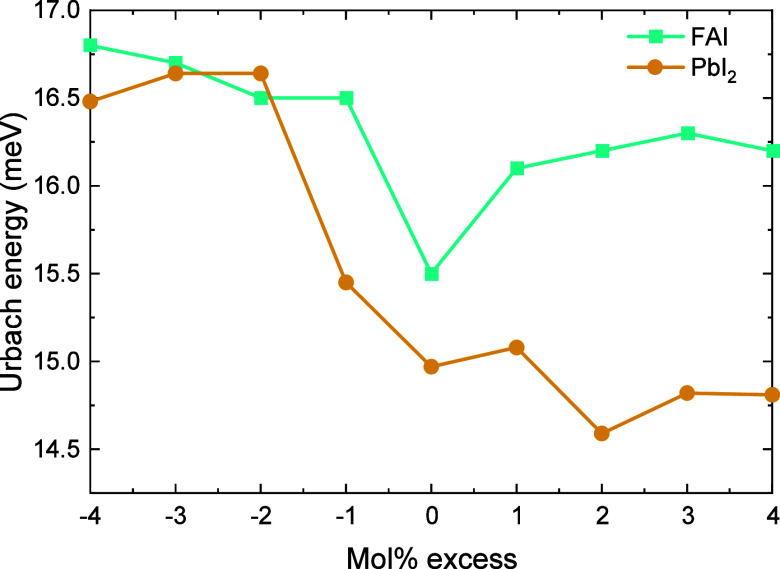
Urbach energies recorded from sub-bandgap EQE of ITO|NiO_*x*_|Me-4PACz|Al_2_O_3_|Cs_0.2_FA_0.8_Pb­(I_0.6_Br_0.4_)_3_|PDAI_2_|PCBM|BCP|Ag solar cells processed from precursor solutions with stoichiometric compositions or with an excess or deficiency of PbI_2_ or FAI.

## 4 Conclusions

The present study shows that it is essential to maintain the ABX_3_ stoichiometry when producing perovskite solar cells. Using a wide-bandgap (1.77 eV) Cs_0.2_FA_0.8_Pb­(I_0.6_Br_0.4_)_3_ perovskite, we find that adding low concentrations (0.5 to 2 mol %) of commonly used lead-salt additives (PbCl_2_ and Pb­(SCN)_2_) to the precursor solution disturbs the stoichiometry of the perovskite absorber. The presence of excess Pb^2+^ leads to drastic reduction of device performance. This detrimental effect can be circumvented by simultaneously providing excess FAI to restore the stoichiometry to its original ABX_3_ composition. Interestingly, the amount of FAI needed differs with lead-salt anion. Because Cl^–^ is incorporated into the crystal lattice and SCN^–^ is not, different amounts of FAI are required to restore the ABX_3_ stoichiometry. For PbCl_2_, adding one equivalent of FAI leads to the formation of stoichiometric FAPb­(Cl_0.66_I_0.33_)_3_ and reinstates the optimized device performance, whereas Pb­(SCN)_2_ requires three equivalents of FAI to compensate the formation of excess PbI_2_, which has to be converted into a FAPbI_3_ stoichiometry and replace the formamidine that is lost during thermal annealing along with thiocyanic acid. While this study mainly focuses on lead-based additives, the stoichiometry-driven mechanism, by which additives affect the perovskite absorber composition, will likely also apply to lead-free additives (such as, e.g., NH_4_Cl, NH_4_SCN, FACl) as demonstrated from the analogous effects observed when creating an excess or deficiency of FAI.

Disturbing the stoichiometry of a perovskite absorber by a deliberate excess or deficiency of either FAI or PbI_2_ in the precursor solution results in similar behavior and a slight disturbance has significant consequences for device performance. A FAI excess leads to detrimental changes in performance as a PbI_2_ deficiency and vice versa. When subsequently adding an ETL we showed that, from a disturbed stoichiometry, the excess FAI migrates toward the top surface of the perovskite and significantly reduces interfacial nonradiative recombination losses, but does not result in improved device performance because of ionic accumulation and impeded charge extraction.

This study serves as a foundation for further research on additives, by emphasizing the importance of maintaining stoichiometry within the absorber.

## Supplementary Material



## Notes

Contains additional *J–V* characteristics, SEM images, box plots with hysteresis index, QFLS data and XPS, UV/vis, PL, and EQE spectra (PDF)
