# Supplementary material for: The Importance of Conserving the Stoichiometry of Wide-Bandgap Perovskites in Additive Engineering
Source: ACS Appl Energy Mater. 2025 Sep 17;8(19):14486–97. doi: 10.1021/acsaem.5c02216 (PMC12522091; doi:10.1021/acsaem.5c02216)
Supplement: Supplementary file 1 [file ae5c02216_si_001.pdf]

Supporting Information

The Importance of Conserving the Stoichiometry of Wide-Bandgap Perovskites in Additive Engineering

Nick R. M. Schipper,[1] Guus J. W. Aalbers,[1] Laura Bellini,[1] Simon V. Quiroz Monnens,[1] Lana M. Kessels,[1] Junke Wang,[2] Martijn M. Wienk,[1] and René A. J. Janssen[1,3]*

[1] Molecular Materials and Nanosystems & Institute for Complex Molecular Systems, Eindhoven University of Technology, P.O. Box 513, 5600 MB Eindhoven, The Netherlands

[2] Clarendon Laboratory, Department of Physics, University of Oxford, OX1 3PU, Oxford, United Kingdom

[3] Dutch Institute for Fundamental Energy Research, De Zaale 20, 5612 AJ Eindhoven, The Netherlands

Corresponding Author

* r.a.j.janssen@tue.nl

Supplementary Figures S1 – S18

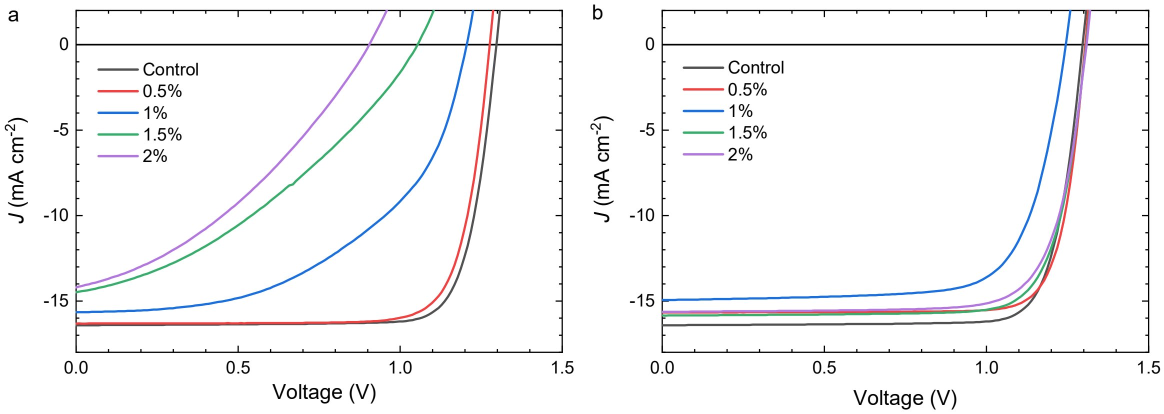

Figure S1. Representative current density – voltage characteristics of ITO|NiO_x|Me-4PACz|Al_2O_3|$Cs_{0.2}FA_{0.8}Pb(I_{0.6}Br_{0.4})_3$|$PDAI_2$|PCBM|BCP|Ag solar cells processed without and with different mol% of $Pb(SCN)_2$ (a) or $PbCl_2$ (b) in the precursor solution. No additional FAI was used.

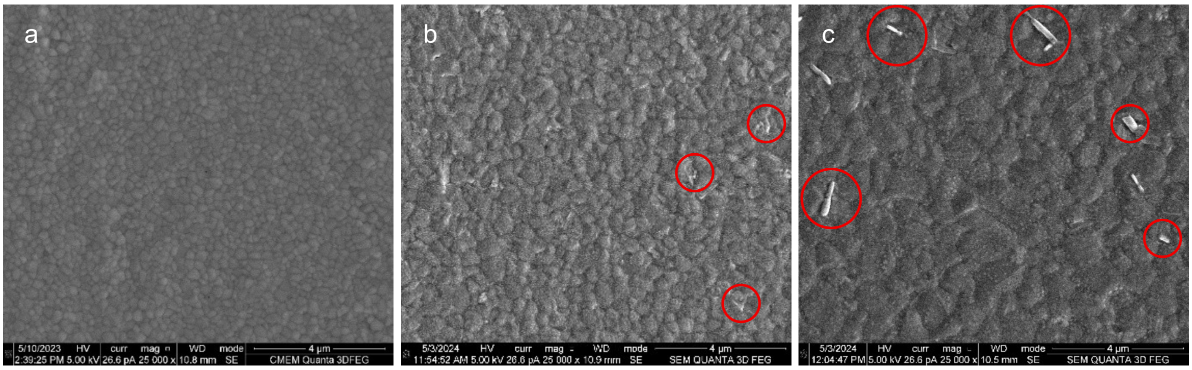

Figure S2. SEM images of $Cs_{0.2}FA_{0.8}Pb(I_{0.6}Br_{0.4})_3$ perovskite films processed with different amounts of $Pb(SCN)_2$ in the precursor solution. a) 0.5 mol%. b) 1 mol%. c) 2 mol%. No additional FAI was used. Note that the grain size increases by adding the additive and that many small and some larger crystallites appear at the surface when increasing the amount of additive. Scale bar is 4 µm. Excess PbI_2 has been circled in red.

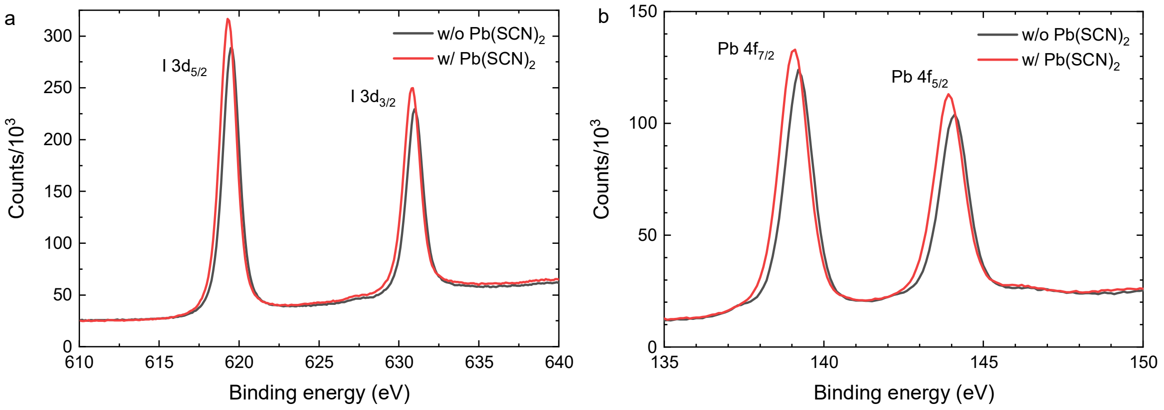

Figure S3. a) I 3d and b) Pb 4f XPS surface scans of a $Cs_{0.2}FA_{0.8}Pb(I_{0.6}Br_{0.4})_3$ perovskite films processed without and with 5 mol% of $Pb(SCN)_2$. With $Pb(SCN)_2$ the surface concentrations of iodide and lead are increased. No additional FAI was used.

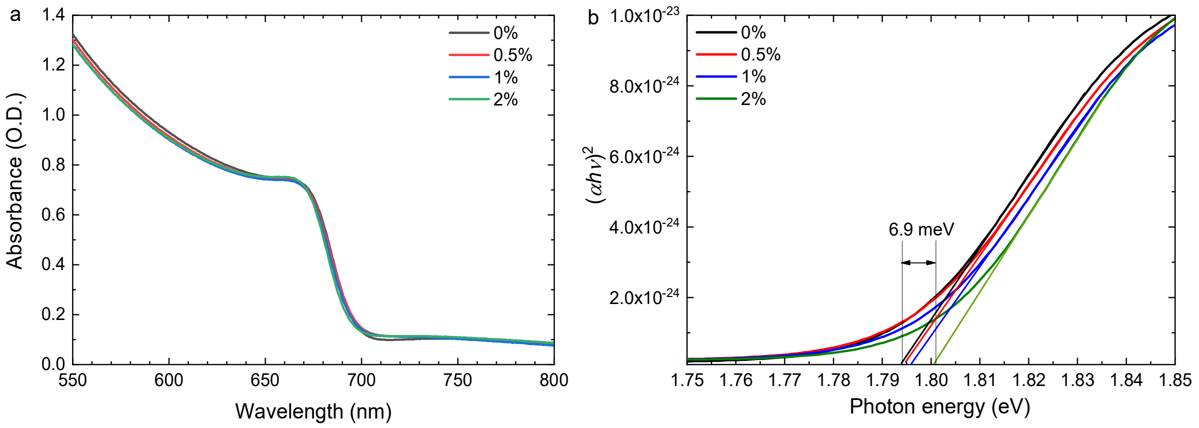

Figure S4. a) UV-vis spectra and b) Tauc plots of $Cs_{0.2}FA_{0.8}Pb(I_{0.6}Br_{0.4})_3$ perovskite films processed with 0 – 2 mol% of $Pb(SCN)_2$ as additive in the solution. No additional FAI was used.

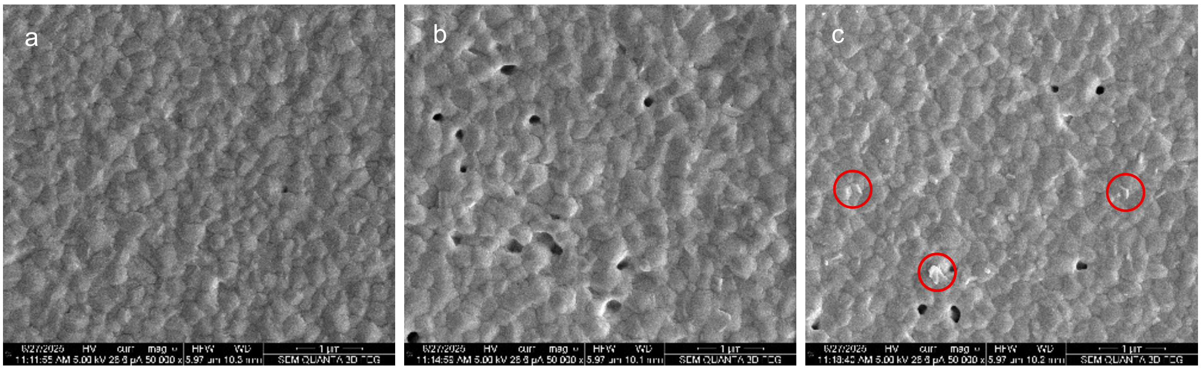

Figure S5. SEM images of $Cs_{0.2}FA_{0.8}Pb(I_{0.6}Br_{0.4})_3$ perovskite films processed with different amounts of $PbCl_2$ in the precursor solution. a) 0.5 mol%. b) 1 mol%. c) 2 mol%. No additional FAI was used. Scale bar is 4 µm. Excess PbI_2 has been circled in red.

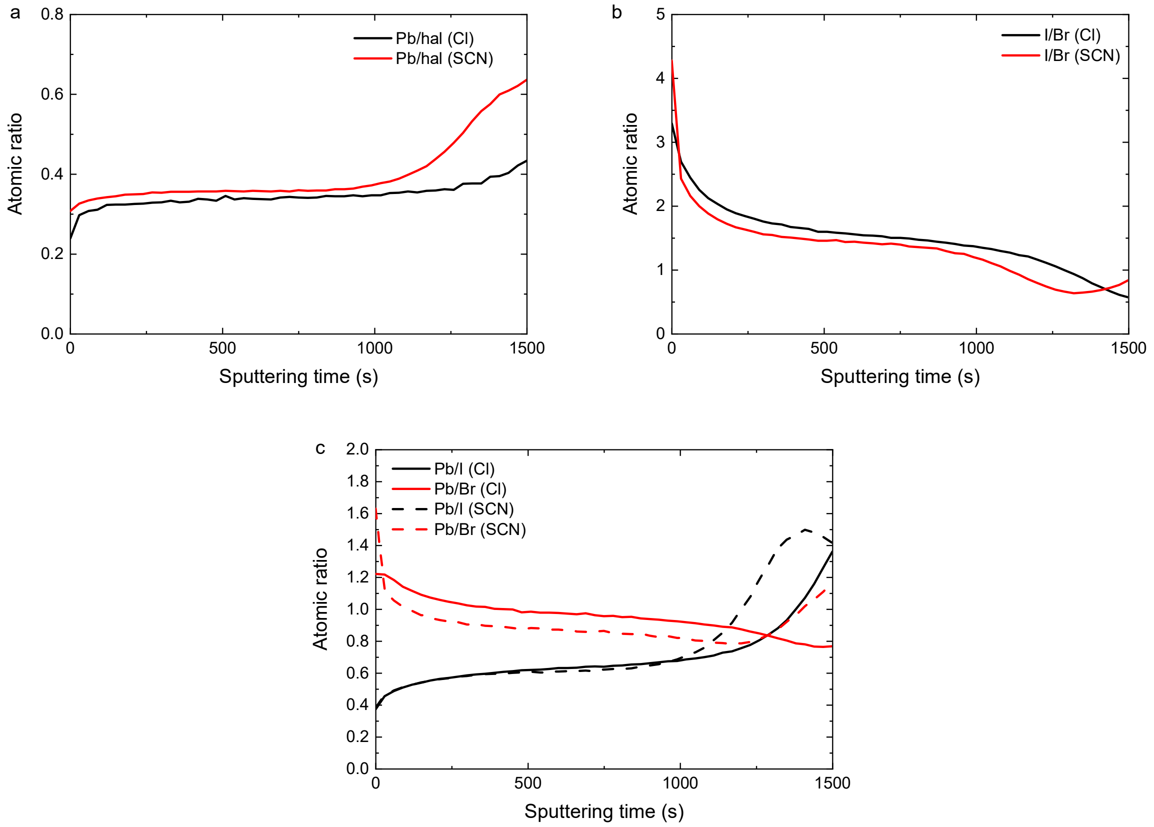

Figure S6. Elemental atomic ratios obtained from XPS-depth scanning of $Cs_{0.2}FA_{0.8}Pb(I_{0.6}Br_{0.4})_3$ perovskite films processed with 5 mol% of $Pb(SCN)_2$ or $PbCl_2$. a) Lead-halide ratio. b) Iodide-bromide ratio. c) Lead-iodide and lead-bromide ratios.

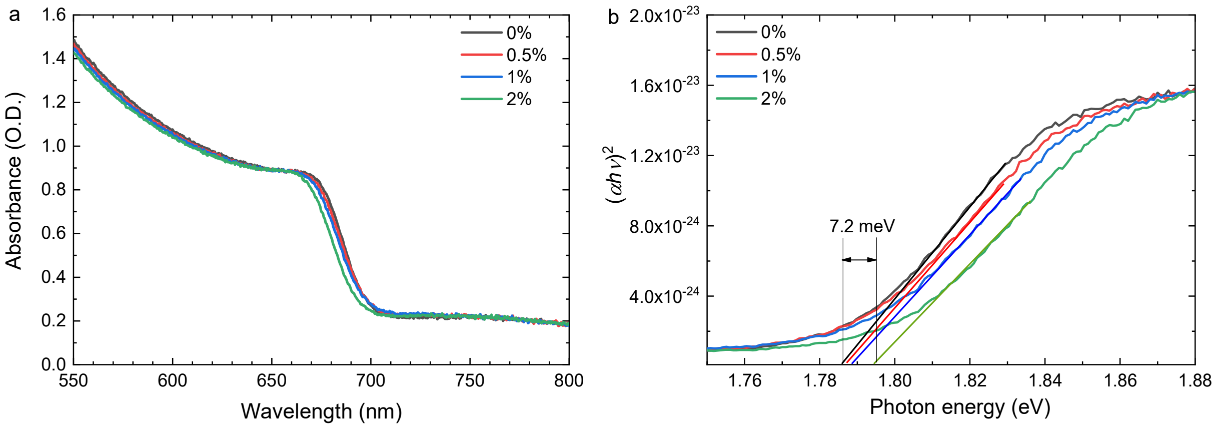

Figure S7. a) UV-vis spectra and b) Tauc plots of $Cs_{0.2}FA_{0.8}Pb(I_{0.6}Br_{0.4})_3$ perovskite films processed with 0 – 2 mol% of $PbCl_2$ as additive in the solution. No additional FAI was used.

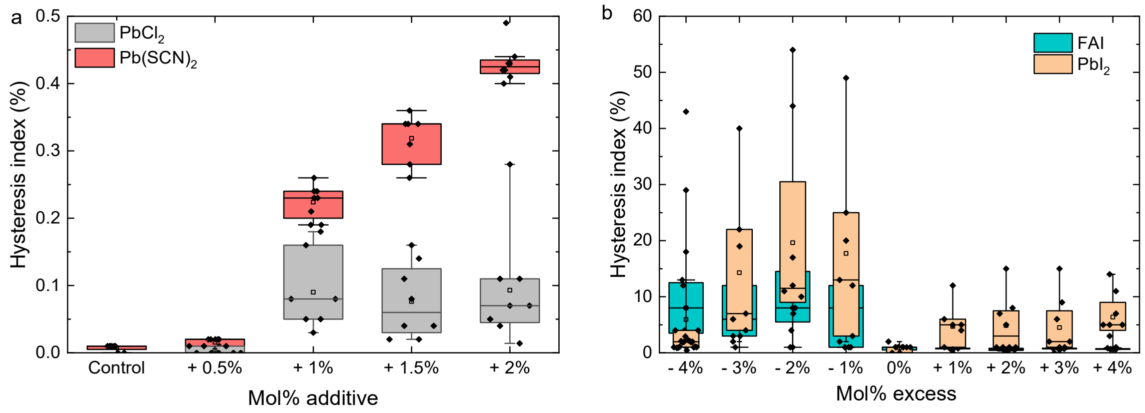

Figure S8. Hysteresis index (HI) of forward and reverse current density – voltage characteristics of ITO|NiO$_x$|Me-4PACz|Al_2O_3|$Cs_{0.2}FA_{0.8}Pb(I_{0.6}Br_{0.4})_3$|$PDAI_2$|PCBM|BCP|Ag solar cells processed without and with different mol% of $Pb(SCN)_2$ or $PbCl_2$ in the precursor solution without excess FAI (a) or a FAI or PbI_2 deficiency or excess (b). The HI was calculated as HI = 100% × (PCE(reverse) − PCE(forward)) / PCE(reverse). The boxplots show the mean (open square), median (center line), 25th and 75th percentiles (box limits), and minimum and maximum (whiskers).

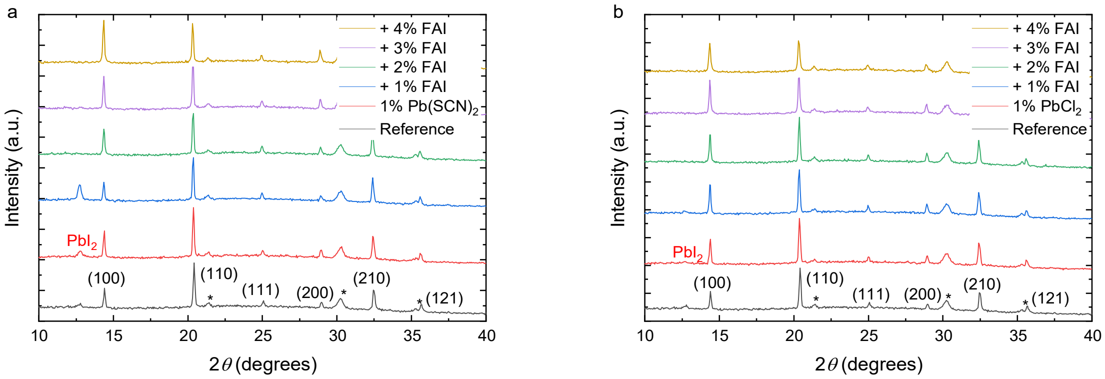

Figure S9. X-ray diffractograms of $Cs_{0.2}FA_{0.8}Pb(I_{0.6}Br_{0.4})_3$ perovskite films processed with a) $Pb(SCN)_2$ and b) $PbCl_2$, alongside with 0 – 4 mol% of excess FAI. The reference film has no additive in the precursor solution. Peaks were assigned by assuming a cubic unit cell in the space group $Pm\overline{3}m$. Peaks indicated with an asterisk are from ITO.

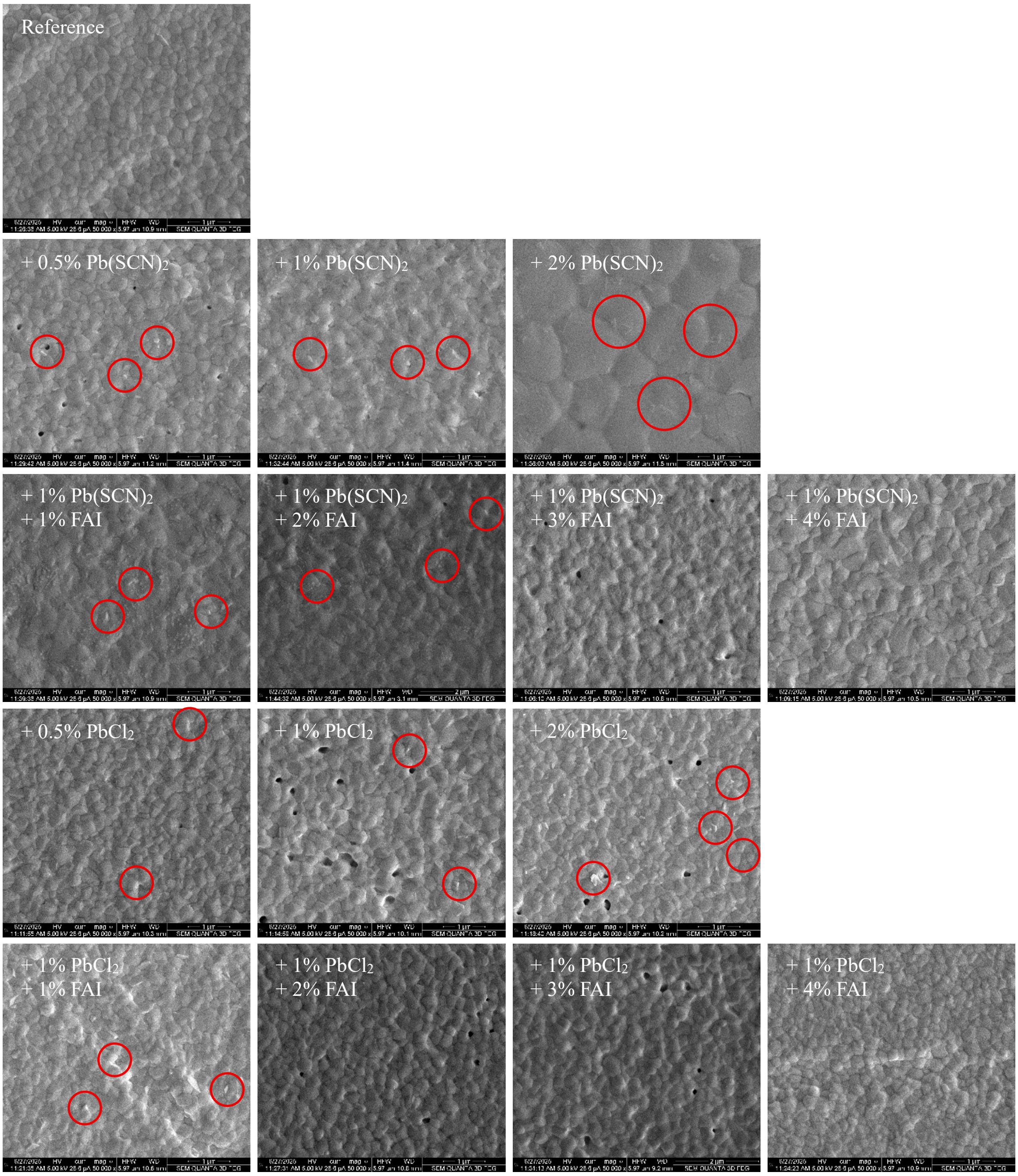

Figure S10. SEM images of $Cs_{0.2}FA_{0.8}Pb(I_{0.6}Br_{0.4})_3$ perovskite films made from: stoichiometric precursor solution (top row), precursors alloyed with 0.5-2 mol% of $Pb(SCN)_2$ (second row), precursors alloyed with 1 mol% of $Pb(SCN)_2$ and 1-4 mol% of excess FAI (third row), precursors alloyed with 0.5-2 mol% of $PbCl_2$ (fourth row), and precursors alloyed with 1 mol% of $PbCl_2$ and 1-4 mol% of excess FAI (fifth row). Examples of excess PbI_2 have been circled in red.

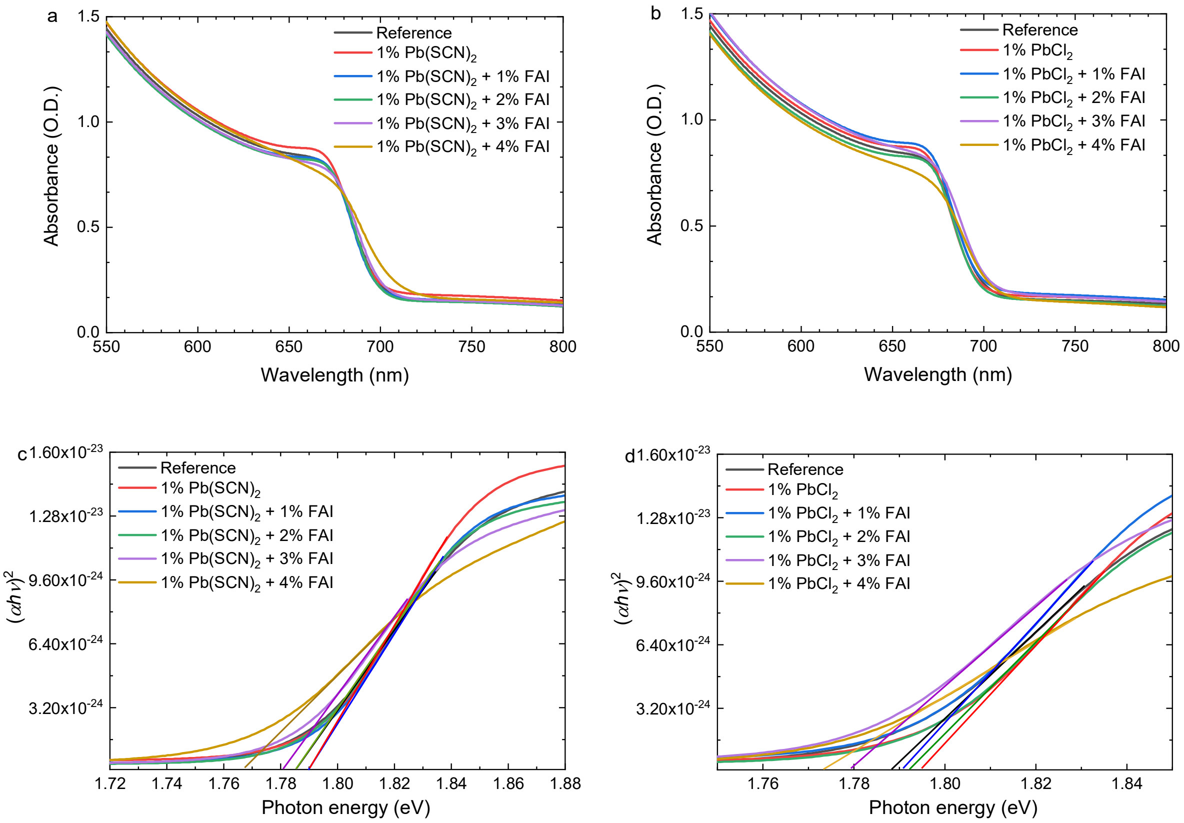

Figure S11. (a, b) UV-vis spectra and (c, d) Tauc plots of $Cs_{0.2}FA_{0.8}Pb(I_{0.6}Br_{0.4})_3$ perovskite films processed with 1 mol% of (a, c) $Pb(SCN)_2$ or (b, d) $PbCl_2$ as additive in the solution, along with 0 – 4 mol% of excess FAI.

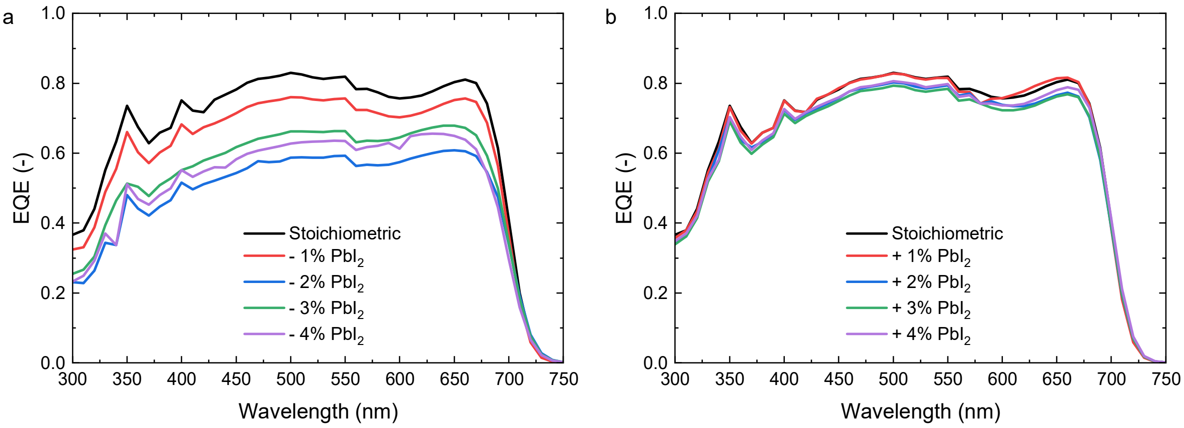

Figure S12. EQE spectra of ITO|NiO_x|Me-4PACz|Al_2O_3|$Cs_{0.2}FA_{0.8}Pb(I_{0.6}Br_{0.4})_3$|$PDAI_2$|PCBM|BCP|Ag solar cells processed from precursor solutions with stoichiometric compositions or with (a) a deficiency or (b) an excess of PbI_2.

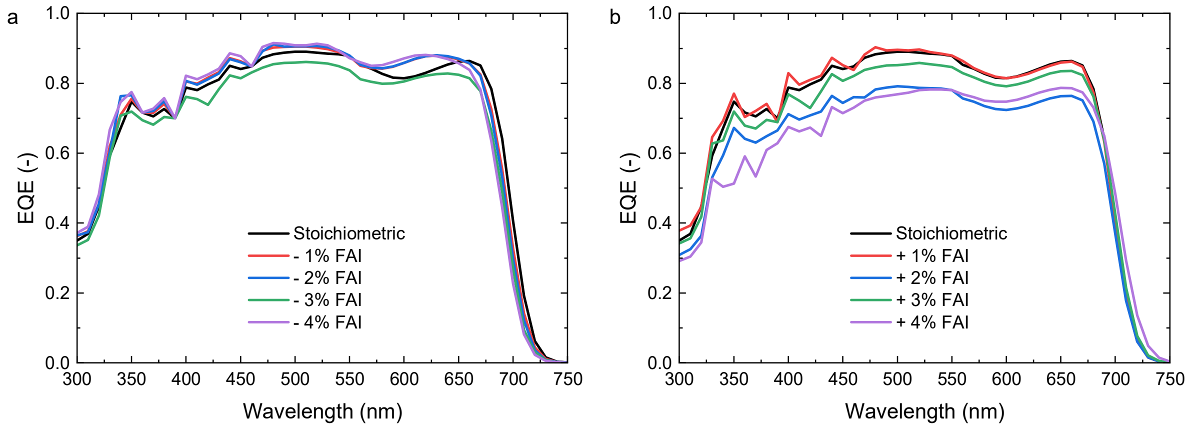

Figure S13. EQE spectra of ITO|NiO_x|Me-4PACz|Al_2O_3|$Cs_{0.2}FA_{0.8}Pb(I_{0.6}Br_{0.4})_3$|$PDAI_2$|PCBM|BCP|Ag solar cells processed from precursor solutions with stoichiometric compositions or with (a) a deficiency or (b) an excess of FAI.

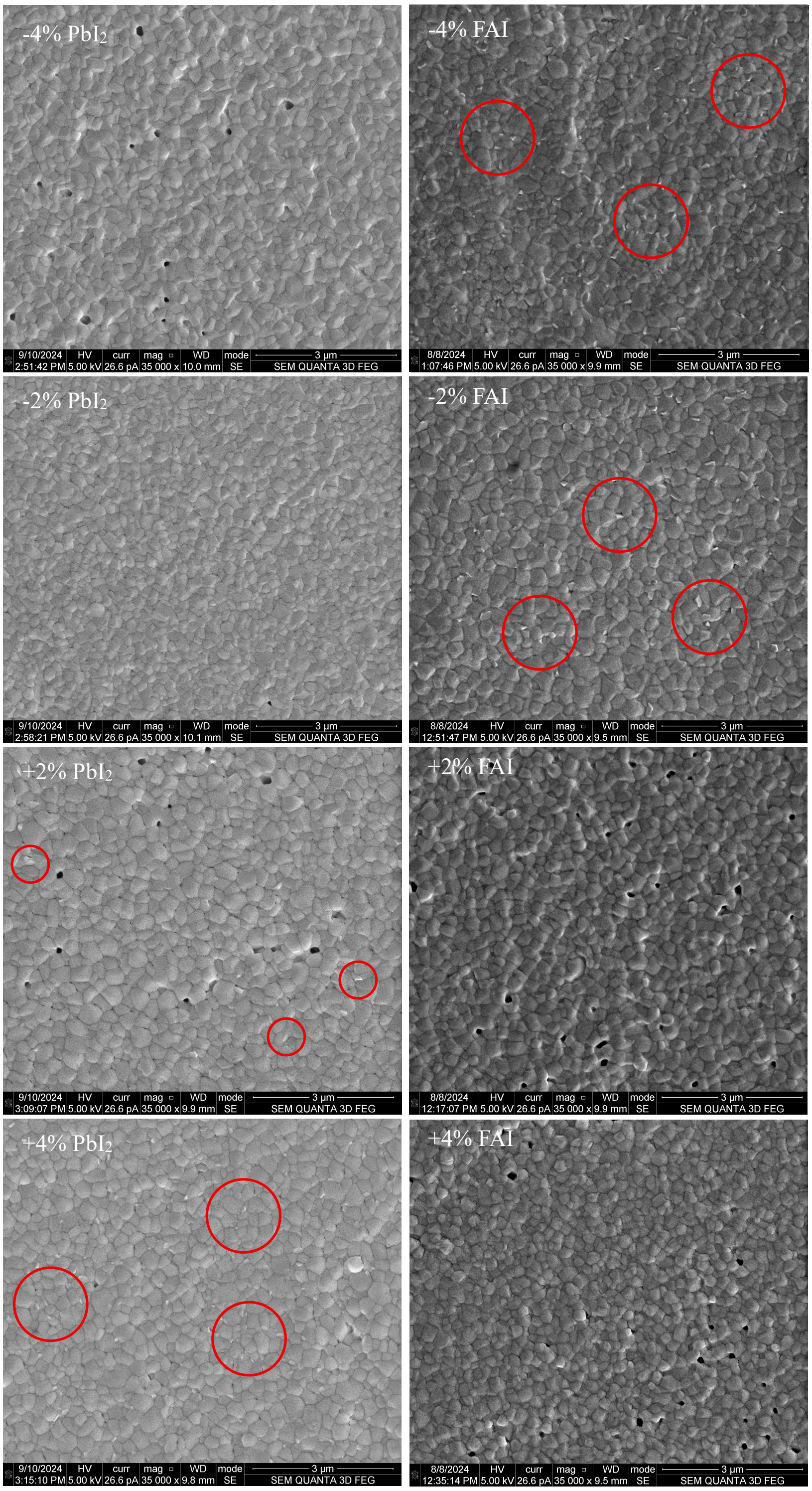

Figure S14. SEM images of $Cs_{0.2}FA_{0.8}Pb(I_{0.6}Br_{0.4})_3$ perovskite films processed from precursor solutions with -4, -2, +2 and +4 mol% excess of PbI_2 and FAI. Examples of excess PbI_2 have been circled in red.

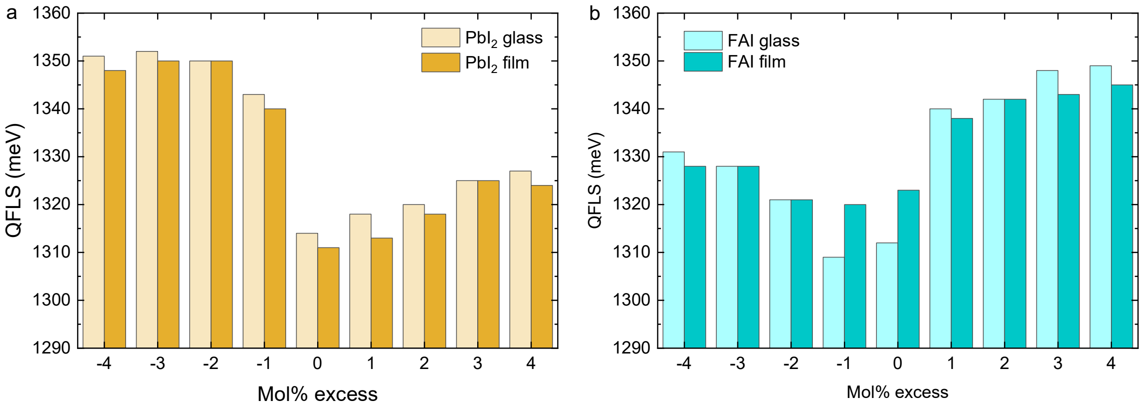

Figure S15. QFLS determined from absolute photoluminescence when measured from the glass or film side for glass|ITO|HDPA|$Cs_{0.2}FA_{0.8}Pb(I_{0.6}Br_{0.4})_3$ samples processed with an excess or deficiency of PbI_2 (a) or FAI (b) in the precursor solution.

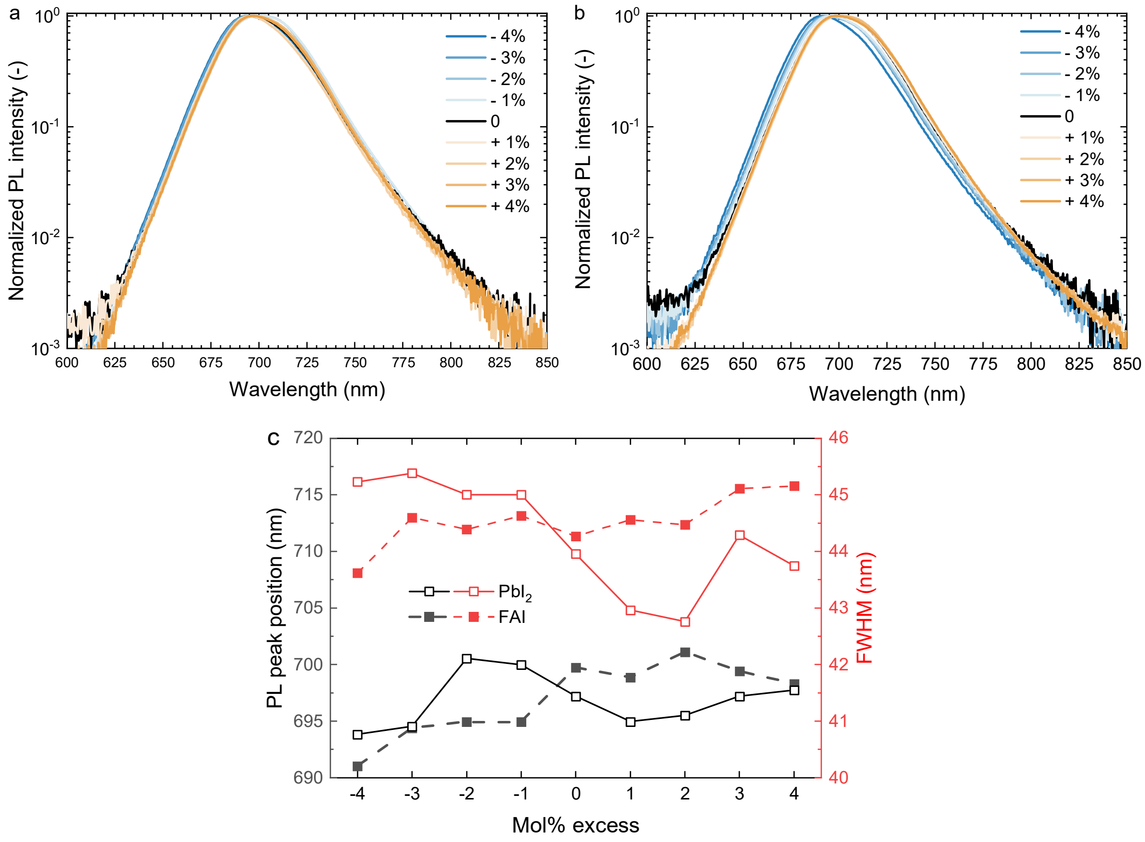

Figure S16. Normalized PL for glass|ITO|HDPA|$Cs_{0.2}FA_{0.8}Pb(I_{0.6}Br_{0.4})_3$ samples processed with excess or deficiency of PbI_2 (a) or FAI (b) in the precursor solution. (c) Evolution of PL peak position and its full width at half maximum (FWHM) vs. molar excess.

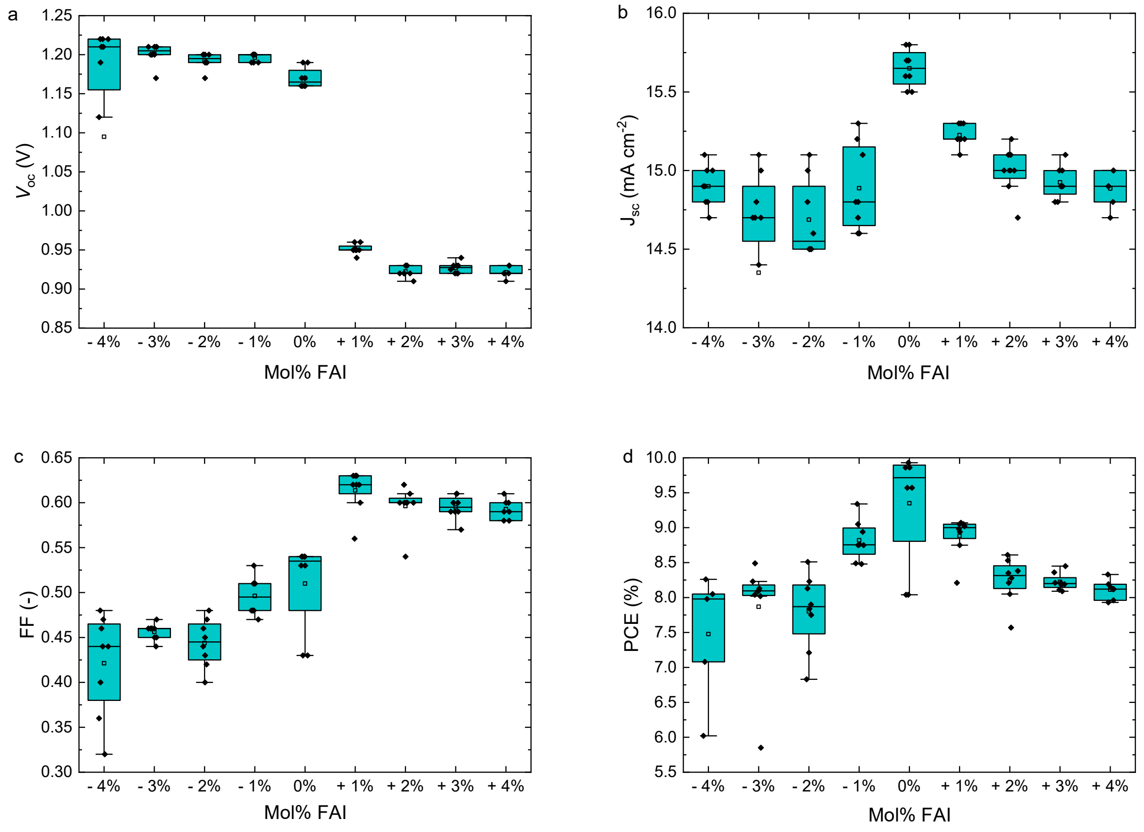

Figure S17. Boxplots of the photovoltaic parameters a) V_{oc}, b) J_{sc}, c) FF, and d) PCE, of ITO|NiO_x|Me-4PACz|Al_2O_3|$Cs_{0.2}FA_{0.8}Pb(I_{0.6}Br_{0.4})_3$|PCBM|BCP|Ag solar cells (8 devices per variation, measured as reverse scan) processed with −4 to +4 mol% of FAI in the precursor solution. The boxplots show the mean (open square), median (center line), 25th and 75th percentiles (box limits), and minimum and maximum (whiskers). 0% mol excess represents fully stoichiometric films.

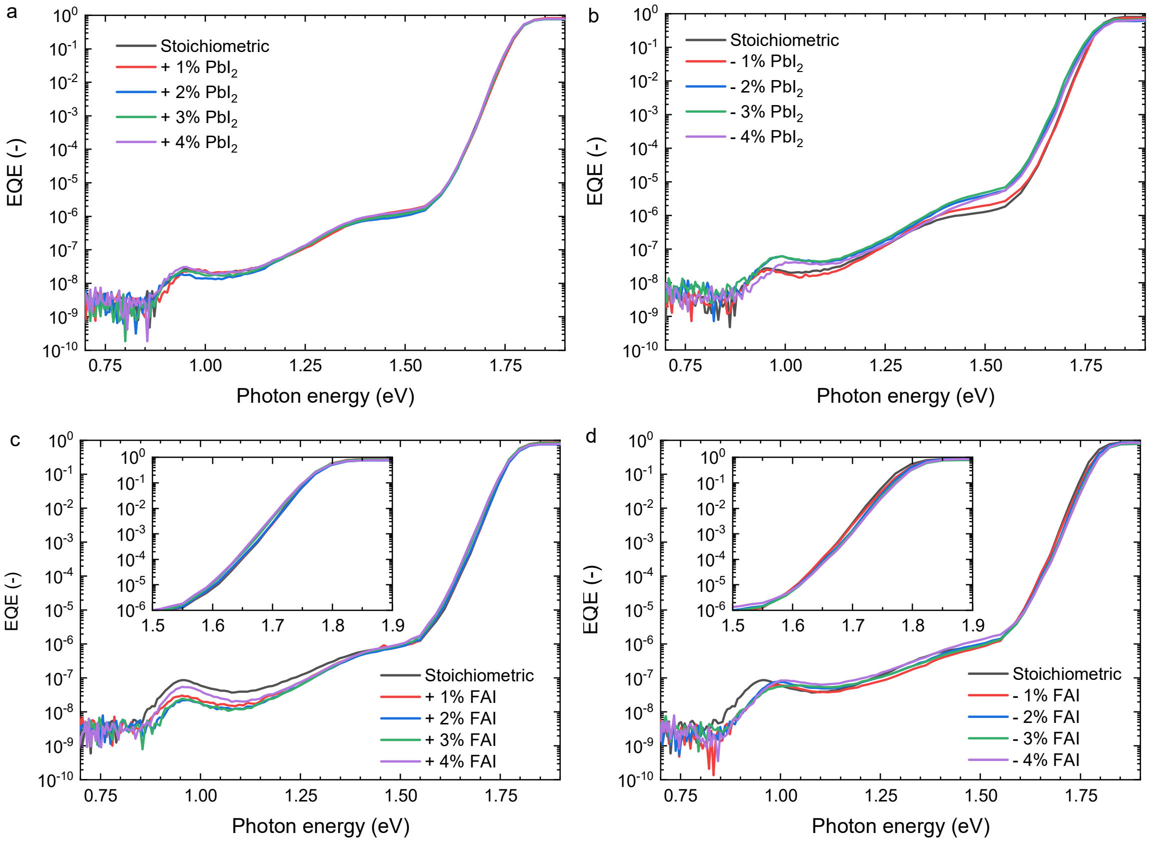

Figure S18. Highly sensitive EQE spectra of ITO|NiO_x|Me-4PACz|Al_2O_3|$Cs_{0.2}FA_{0.8}Pb(I_{0.6}Br_{0.4})_3$|$PDAI_2$|PCBM|BCP|Ag solar cells processed from precursor solutions with stoichiometric compositions or with an excess (a, c) or deficiency (b, d) of PbI_2 (a, b) or FAI (c, d).
